# Prediction of Clinically Meaningful Improvement After Internet-Delivered Cognitive Behavioral Therapy for Depression and Anxiety Disorders: Machine Learning–Based Predictive Model Development and Temporal Validation Study

**DOI:** 10.2196/100162

**Published:** 2026-08-07

**Authors:** Olly Kravchenko, Matthew Halvorsen, Julia Bäckman, Viktor Kaldo, James J Crowley, Ralf Kuja-Halkola, Christian Rück, John Wallert

**Affiliations:** 1 Centre for Psychiatry Research, Department of Clinical Neuroscience, Karolinska Institutet, & Stockholm Health Care Services, M48, Karolinska Universitetssjukhuset Huddinge, Region Stockholm, Stockholm, 14186, Sweden, 46 709604983; 2 Department of Genetics, University of North Carolina at Chapel Hill, Chapel Hill, NC, United States; 3 Department of Psychology, Faculty of Health and Life Sciences, Linnaeus University, Växjö, Sweden; 4 Department of Medical Epidemiology and Biostatistics, Karolinska Institutet, Stockholm, Sweden

**Keywords:** internet-delivered cognitive behavioral therapy, digital mental health, machine learning, treatment outcome prediction, baseline prediction, depression, anxiety, polygenic scores, precision psychiatry

## Abstract

**Background:**

Up to 50% of patients treated with internet-delivered cognitive behavioral therapy (ICBT) for depression and anxiety disorders do not experience clinically significant symptom reduction. Identifying these patients prior to the initiation of ICBT can support treatment planning.

**Objective:**

The aim of this study was to enhance baseline prediction of clinically meaningful improvement in patients treated with ICBT for common psychiatric disorders in routine care, which could ultimately inform treatment planning at intake.

**Methods:**

We developed multimodal predictive models integrating clinical, sociodemographic, and genetic data available pretreatment to predict clinically meaningful improvement in a sample of 1790 patients treated with ICBT for major depressive disorder, panic disorder, and social anxiety disorder. We applied machine learning algorithms of varying complexity (logistic regression, random forest [RF], extreme gradient boosting, support vector machines, soft voting, and stacking ensemble), with nested cross-validation, elastic net variable selection, multiple imputation, and temporal validation in a 20% holdout test set (n=356). The primary performance measure was the area under the receiver operating characteristic curve (AUC).

**Results:**

All full phenotypic models showed comparable performance (AUC_test_ 0.732‐0.749), with RF achieving the best holdout discrimination (AUC_test_ 0.749, 95% CI 0.698-0.797). Compared with the benchmark model based on self-reported screening data (AUC_test_ 0.695, 95% CI 0.637-0.748), RF and both ensemble models incorporating register data showed higher discrimination in paired DeLong tests (*P*=.04, *P*=.02, and *P*=.03, respectively), whereas polygenic scores added no independent predictive value in this cohort and modeling setup (*P*=.97).

**Conclusions:**

These promising results support the feasibility of baseline prognostic prediction of clinically meaningful improvement after ICBT and provide a basis for the prospective validation of model-informed risk stratification.

## Introduction

Cognitive behavioral therapy (CBT) is a first-line treatment for mild-to-moderate depression and anxiety disorders [1]. To expand treatment coverage, enhance flexibility, and reduce costs, CBT is increasingly delivered remotely. Existing evidence suggests that internet-delivered CBT (ICBT) is comparable to face-to-face CBT in reducing symptom severity, expands access to mental health services by mitigating patients’ financial and time constraints, and requires less therapist time, thereby increasing patient throughput and lowering health care costs [2-5]. However, similar to traditional CBT, up to 50% of patients do not experience clinically significant improvement [6-8] and are at risk of clinical deterioration and poor long-term outcomes [9].

Numerous studies have sought to identify robust predictors of treatment outcome to facilitate patient stratification. Existing evidence suggests several clinical (baseline symptom severity [6,10-12] and comorbidity [6,10,12]) and sociodemographic (education [10,11] and employment [10,13,14]) predictors of CBT outcome; however, no reliable molecular or neuroimaging biomarkers have yet been identified [15]. Recent advancements in psychiatric genomics support a substantial contribution of genetic differences to the variance in complex traits, including the onset and prognosis of psychiatric disorders [16]. Genome-wide association studies (GWAS) provide data on population-level associations of single nucleotide polymorphisms (SNPs) and phenotypes of interest. SNPs are commonly aggregated into polygenic scores (PGSs), which summarize small effects of multiple risk alleles on a specific trait. Therapygenetics is an area of research that specifically aims to quantify the contribution of genetic variation to psychotherapeutic treatment outcomes [17]. Yet, the 3 existing GWAS of CBT response did not identify any genome-wide significant loci and failed to derive stable SNP-based heritability estimates, possibly due to insufficient sample sizes (n=980‐3113) [18-20]. However, there are some indications that common genetic variants may be implicated in CBT response variability. Preliminary findings include a positive association between PGS for educational attainment and symptom reduction [21], a negative association between PGS for autism spectrum disorder and symptom reduction [22], and a weak predictive effect of PGS for depression and intelligence on remission [11].

Despite some advances in identifying group-level predictors, it remains unclear whether they can be effectively translated into meaningful predictions at the individual patient level. Machine learning (ML) has increasingly been applied to predict a future outcome for a yet unobserved individual patient by first training a model using the abundance of retrospective data from other patients. Given evidence that models trained on multiple data types typically outperform single-modality models [23], it has been recommended that future efforts focus on multimodal prediction [24,25]. The premise that justifies the deployment of predictive models in routine psychiatric care is that they add value beyond clinical judgment, which has been shown to be affected by bias and overoptimism [26,27]. Due to a growing interest in precision medicine, new prediction tools emerge continuously; however, most are not adopted clinically, mainly due to low accuracy and lack of external validation. Systematic reviews and meta-analyses of ML studies predicting treatment outcome [28-31] report varying results but emphasize that insufficient sample size lies at the core of inflated performance and poor generalization. In their comprehensive review, Sajjadian et al [28] highlight a strong negative relationship between study quality, defined by sufficient sample size and robust validation methods, and predictive accuracy. The authors raise a concern that the overly optimistic results reported by most reviewed papers stem from insufficient methodological scrutiny rather than genuinely high predictability. Finally, implementation studies applying and assessing predictive models in real-world clinical practice are scarce, demonstrating a substantial translational gap [29,32]. In summary, despite the promise of ML approaches, translation to reliable individual-level predictions has been hampered by several persistent gaps: small sample sizes, single data types, and methodological flaws.

The aim of this study was to enhance the baseline prediction of clinically meaningful improvement in patients treated with ICBT for common psychiatric disorders in routine care. To this end, we integrated multimodal data (clinical, sociodemographic, and genetic) to develop and validate predictive models, which could ultimately inform treatment planning at intake. To address common quality concerns, we applied a rigorous model development framework, including nested cross-validation (CV), multiple imputation (MI), variable selection with complete separation between training and validation sets, and a temporally separated holdout test set. Relative to prior work, distinct contributions of this study are a large real-world routine-care cohort, baseline-only prediction, and the integration of multiple data modalities, including national register linkage and genetic data.

## Methods

### Reporting Guidelines and Preregistration

We followed the PROBAST (Prediction Model Risk of Bias Assessment Tool) [33] and TRIPOD+AI (Transparent Reporting of a Multivariable Prediction Model for Individual Prognosis or Diagnosis+AI) guidelines [34] when conducting analyses and reporting findings. The study was preregistered on the Open Science Framework [35]. Minor changes made to the preregistered protocol are listed in Multimedia Appendix 1.

### Ethical Considerations

This study was approved by the Stockholm Regional Ethical Review Board (REPN 2009/1089-31/2 and 2014/1897‐31) and adhered to the Declaration of Helsinki. All participants provided written informed consent before entering the study. Data were pseudonymized prior to being used for research purposes in accordance with Swedish regulations.

### Sample and Treatment

This study used data from the MULTI-PSYCH cohort comprising 2668 patients treated with ICBT for depression (n=1300), panic disorder (PD; n=727), or social anxiety disorder (SAD; n=641) at the Internet Psychiatry Unit of the Psychiatric Clinic Southwest at Karolinska University Hospital, Huddinge, Sweden, between 2008 and 2020 [36]. All treatments were provided over 12 weeks with therapist guidance via asynchronous messaging and consisted of similarly structured psychoeducation and exercise modules, with disorder-specific differences in intervention content (eg, behavioral activation for depression and exposure therapy for anxiety disorders). During screening, patients completed multiple questionnaires and answered interview questions about their symptomatology, medical history, and socioeconomic status, as well as provided blood or saliva samples for DNA analysis. Each patient’s unique Swedish personal identity number was used for record linkage across multiple nationwide registers: the National Patient Register [37], the Stockholm regional health care database VAL [38], the National Prescribed Drug Register [39], and the LISA (Longitudinal Integrated Database for Health Insurance and Labor Market Studies), which is managed by Statistics Sweden and combines data from the Register-based Employment Statistics, the Total Population Register, the Education Register, the Register of Income and Taxation, the Swedish Public Employment Service, and the Swedish Social Insurance Agency [40].

Given the substantial etiological and pathological overlap among internalizing disorders, treatment similarities, and a growing interest in transdiagnostic clinical prediction tools, the 3 disorder-specific subsamples were combined into a single dataset.

### Predictors

We constructed 2 sets of predictors—phenotypic and genetic—and combined their predicted probabilities via ensembling to produce the final model. The phenotypic set included variables collected during pretreatment screening and from national registers. To enable baseline prediction, we restricted variables to those available before treatment initiation. First, to reduce the initial predictor space, we performed partial a priori selection of candidate predictors informed by empirical evidence and domain expertise [29,41]. Upon manually screening over 1000 variables, we retained 56 phenotypic predictors (Multimedia Appendix 2), which were subsequently refined using elastic net variable selection. For genetic predictors, we employed the approach suggested by Albiñana et al [42], who leveraged high pleiotropy of complex traits and overlapping underlying mechanisms to demonstrate that incorporating hundreds of PGSs for psychiatric, neurological, and behavioral phenotypes can increase polygenic predictions compared to single-phenotype PGS. Following this methodology, we constructed a library of 615 traits, comprising 15 PGSs from the latest disorder-specific psychiatric GWAS (Multimedia Appendix 3) and 600 PGSs from the UK Biobank GWAS Atlas that covers sociodemographic, lifestyle, and health-related traits [16]. Genotyping, imputation, and quality-control procedures are described elsewhere [43]. PGSs were constructed using PRS-CS, a Bayesian polygenic prediction method that places continuous shrinkage priors on SNP effect sizes and improves performance over traditional approaches [44].

### Outcome

Symptom severity was estimated with the Montgomery-Åsberg Depression Rating Scale, Self-Rating Version (MADRS-S) for depression [45], Panic Disorder Severity Scale, Self-Report (PDSS-SR) for PD [46], and Liebowitz Social Anxiety Scale, Self-Report (LSAS-SR) for SAD [47]. The primary outcome was a binary *Clinically meaningful improvement*, coded as 1 if either of the following criteria was met: (1) symptom reduction from pretreatment to posttreatment (≥40% for depression and PD and ≥30% for social anxiety) or (2) remission (MADRS-S≤10, PDSS-SR≤7, and LSAS-SR≤35). The composite outcome was chosen to capture 2 clinically relevant pathways to a favorable treatment outcome: symptom reduction of a magnitude deemed clinically relevant or low posttreatment symptom burden. A remission-only criterion may classify patients as nonimproved despite substantial symptom reduction if they remain above the cutoff level, whereas a large relative change may result in patients being classified as improved despite residual symptoms. Moreover, the remission component of the composite outcome is inherently influenced by baseline severity, as patients with lower pretreatment scores require a smaller absolute reduction to meet a remission cutoff, whereas higher baseline severity may make remission harder despite substantial relative improvement. The composite definition was therefore intended to partially mitigate limitations of both end points and reflect a clinically meaningful positive outcome either by reaching an acceptable end-state symptom level or by improving enough that the treatment outcome can be judged clinically beneficial. The primary binary outcome was constructed from original disorder-specific scales: raw pretreatment and posttreatment scores for relative symptom reduction and raw-score cutoffs for remission. Disorder-specific cutoffs for symptom reduction and remission were applied to account for differences in clinically significant symptom reduction across disorders, following the SibeR (Swedish National Quality Register for internet-based psychological treatment) guidelines and existing literature [48-50]. A binary outcome was chosen because it is more directly interpretable and actionable for clinicians than a continuous posttreatment score.

As a secondary outcome, we analyzed the continuous posttreatment score to reduce the information loss inherent in dichotomization. To enable pooling of the 3 disorder-specific symptom scales, MADRS-S, PDSS-SR, and LSAS-SR scores were linearly rescaled to a 0 to 100 range and combined into a single harmonized symptom severity score.

### Analysis

Data preprocessing was performed in R (version 4.3.1; R Foundation for Statistical Computing) and Python (version 3.10.12). Model training and validation were performed in Python, primarily using scikit-learn [51] (1.5.2), NumPy [52] (2.0.0), and pandas [53] (2.2.3) libraries.

#### Phenotypic Models

Variable correlations were ≤0.6. No zero-variance variables were identified. Predictors with near-zero variance (such as prior psychiatric diagnoses with a category frequency <0.01) were excluded (Multimedia Appendix 4). Samples with missing outcome data (n=525) and genetic data (n=456) were also excluded. The final dataset (N=1790) was split into an 80% training set and a 20% holdout test set, comprising the most recently treated patients per disorder, thus allowing for temporal validation to increase model generalizability [54].

##### Missing Data and Preprocessing

In the analytic dataset (N=1790), per-variable missingness was less than 7%. Since listwise deletion of cases with at least 1 missing value results in substantial loss of information, thereby reducing power and external validity [28], MI is a widely recommended approach for handling missing data in predictive models [28,55,56]. Thus, to maximize sample size for modeling and ensure generalizability to new samples with missing data, we applied MI by chained equations with the package miceforest (version 6.0.3) [57]. Diagnostics supported convergence at 5 iterations by 5 imputed datasets. After MI, numeric variables were scaled with StandardScaler, ordinal categorical variables with OrdinalEncoder, and nominal categorical variables were one-hot encoded.

##### Variable Selection

To derive more parsimonious models, we used elastic net, a regularization method that blends L1 (least absolute shrinkage and selection operator [LASSO]) and L2 (ridge), with CV to tune the penalty strength and L1/L2 mix, enabling the selection of an optimal subset of predictors [58]. For ElasticNetCV hyperparameter tuning, L1 ratios in the range {0.1, 0.5, 1.0} and α values in the range {0.001, 0.01, 0.1, 1, 10, 100} were searched. We also compared different approaches to hyperparameter tuning: providing a prespecified grid of L1/α with 5/10-fold elastic net CV vs letting elastic net internally search a path of α vs adding L1/α to the external hyperparameter grid to allow for co-tuning of the classifier’s hyperparameters with the elastic net’s hyperparameters to yield the best combination. Because external variable selection may improve or harm performance, depending on the classification method, each model’s performance with and without elastic net regularization was compared to select the best approach for each algorithm.

##### Modeling

First, we designed a benchmark model: a simple logistic regression (LR) with no hyperparameter tuning, trained on a subset of 28 easily obtainable predictors, self-reported by patients during screening procedures. Then, we employed a range of algorithms to assess incremental performance improvement compared to the benchmark and trained them on the full set of predictors (56 variables): LR (with hyperparameter tuning), random forest (RF), XGBoost (extreme gradient boosting), and support vector machine (SVM) [59]. In addition, we ensembled the 4 classifiers to correct individual model errors and potentially outperform the constituents [60]: soft voting (averaging predicted probabilities and selecting the class with the highest average) and stacking (an LR meta-model trained on the base models’ predictions to produce final predictions). To prevent the misclassification of the minority class, we adjusted for the slight class imbalance (64%/36%) by balancing class weights in LR, RF, and SVM and by applying the *scale_pos_weight* hyperparameter in XGBoost. For the secondary outcome (posttreatment symptom score), we applied the same procedures but with regression counterparts of the same algorithms.

**Figure 1.**
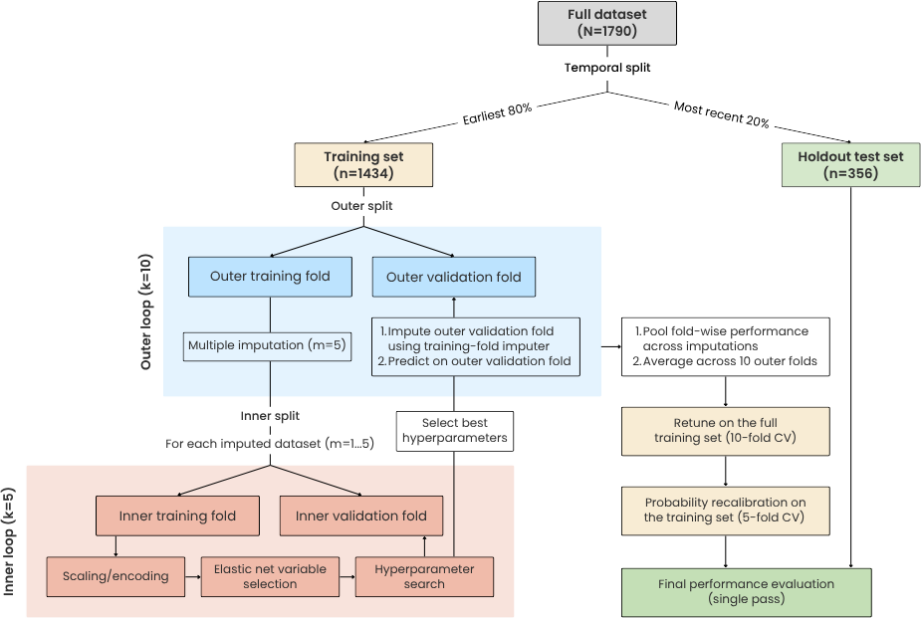
Overview of the modeling pipeline used for model development (left) and evaluation on the holdout test set (right). The full dataset was temporally split into a training set (earliest 80%) and an independent holdout test set (most recent 20%). Within the training set, models were tuned using 5×10 nested cross-validation (CV) with multiple imputation (m=5) and preprocessing performed within folds. Performance estimates were pooled across imputations and averaged across outer folds. Final models were retrained on the full training set and recalibrated before single-pass evaluation on the holdout test set. Elastic net variable selection and hyperparameter search were not applied to the benchmark model.

A summary of the modeling pipeline is shown in Figure 1. To avoid data leakage and reduce overfitting, MI, scaling/encoding, and variable selection were performed within-fold, fully separated between training and validation sets, following the recommended procedure [28]. Nested CV is considered the most robust CV method, as it yields accurate CIs for prediction error and controls for overfitting to a single training/validation split by performing hyperparameter tuning and variable selection in the inner loop and independently estimating model performance in the outer loop [28,61,62]. For all but the benchmark model, we performed nested CV with 5 inner and 10 outer folds. For LR, RF, and SVM, we used grid search for hyperparameter tuning to evaluate all the combinations within a prespecified set of hyperparameters. For XGBoost, we applied Bayesian optimization using the package scikit-optimize (version 0.10.2) [63], which performs iterative hyperparameter sampling, guided by previous results, and is more suitable for a multidimensional hyperparameter space. Original ranges were expanded if the selected hyperparameters were close to the lower or upper bounds. A list of hyperparameters is provided in Multimedia Appendix 5.

##### Evaluation

The area under the receiver operating characteristic curve (AUC) was the primary metric used for model optimization and performance evaluation. To comprehensively capture all aspects of model performance, we also assessed balanced accuracy, prioritized over standard accuracy for its robustness to class imbalance, *F*_1_-score, log loss, and the Matthews correlation coefficient. Final training metrics were pooled across 5 imputed datasets per fold using the Rubin’s rules and then averaged across the 10 outer folds of the nested CV. To ensure the reliability of predicted probabilities, we assessed probability calibration (agreement between predicted and observed values) using calibration curves. To this end, predictions were grouped into deciles of predicted risk and plotted against observed outcome events. After obtaining an unbiased performance estimate across the 10 outer folds of the nested CV, each model was retrained on the entire training set with a new 10-fold CV and evaluated on the same 20% holdout test set (n=356). Finally, all models’ AUC_test_ values were compared with unadjusted paired DeLong tests on the same holdout test set sample. For the secondary analysis of the continuous outcome, performance was evaluated with root mean-squared error (RMSE), mean absolute error (MAE), and variance explained (*R*^2^).

### Genetic Models

To mitigate the risk of overfitting, expert knowledge was applied to filter the original GWAS Atlas of 600 PGSs and preselect those with higher relevance for the ICBT outcome. While based on face validity and theory, this selection was intentionally liberal to allow unexpected associations to emerge. To address collinearity, predictor pairs with a variance inflation factor >5 were excluded. The resulting final set contained a total of 293 PGSs. Following the methodology in Albiñana et al [42], upon standardizing the PGSs, we trained a linear (LASSO‐penalized regression) and a nonlinear model (XGBoost). The hyperparameter tuning procedure mirrored the phenotypic models. Sex, age, and the first 20 principal components were included to control for population stratification, and models with and without covariates were compared by the increment in AUC (ΔAUC). Finally, the model with the best performance across the 10 outer folds of the nested CV was retrained on the entire training set and evaluated on the holdout test set.

### Final Ensemble

Phenotypic and genetic predictor spaces differ substantially in their structure, dimensionality, and signal-to-noise ratio, requiring separate optimization pipelines. Therefore, we used a late fusion strategy to combine predictions from the 2 data modalities. Specifically, out-of-fold predicted probabilities from the best-performing phenotypic and genetic models were combined in an LR stacking meta-learner. This approach was selected because it allows the meta-learner to downweight a constituent model that contributes little independent predictive signal and provides a parsimonious way to estimate the relative contribution of each predictor type. It also enables a direct evaluation of whether genetic predictions add incremental value beyond phenotypic predictions.

### Sensitivity Analyses

#### Complete-Case Analysis

To appraise the impact of MI, all the models were also trained and evaluated using the same pipeline on the complete-case dataset (n=1381) as a sensitivity analysis.

#### Expanded Phenotypic-Only Sample Without the Exclusion of Rows With Missing Genetic Data

The primary analytic sample was restricted to patients with available outcome and genetic data to enable the direct comparison of phenotypic and phenotypic-genetic ensemble models within the same sample and temporal split. Because phenotypic-only prediction does not require genetic data, we conducted a sensitivity analysis including all patients regardless of genetic-data availability (n=2143, training set: n=1716, holdout test set n=427). The same preprocessing, imputation, training, recalibration, and evaluation pipeline was applied as in the main analyses.

## Results

### Sample Characteristics

Key clinical and demographic sample characteristics are summarized in Table 1, with detailed descriptive statistics and comparisons between the training and test samples provided in Multimedia Appendix 6. A total of 1115 (62.3%) patients achieved clinically meaningful improvement, of whom 753 (67.5%) met both the symptom reduction and remission criteria, 258 (23.1%) achieved symptom reduction only, and 104 (9.3%) achieved remission only, illustrating that the 2 criteria capture partially distinct clinically relevant outcomes.

**Table 1.** Key characteristics of the total sample and stratified by disorder.

Variable	Total (N=1790)	Depression (n=858)	Panic disorder (n=469)	Social anxiety (n=463)
Clinically meaningful improvement^a^, n (%)	1115 (62.3)	495 (57.7)	380 (81.0)	240 (51.8)
Both criteria, n (%)	753 (42.1)	331 (38.6)	310 (66.1)	112 (24.2)
Symptom reduction criterion only, n (%)	258 (14.4)	141 (16.4)	19 (4.1)	98 (21.2)
Remission criterion only, n (%)	104 (5.8)	23 (2.7)	51 (10.9)	30 (6.5)
Pretreatment symptom severity^b^, mean (SD)	47.1 (17.3)	51.6 (15.3)	39.8 (18.1)	46.2 (17.3)
Posttreatment symptom severity^b^, mean (SD)	28.1 (18.8)	30.6 (18.7)	17.2 (15.9)	34.5 (16.9)
Age (y), mean (SD)	36.2 (11.6)	38.3 (11.9)	35.1 (11.2)	33.2 (10.7)
Female sex, n (%)	1126 (62.9)	579 (67.5)	277 (59.1)	270 (58.3)
Married, n (%)	1075 (60.1)	499 (58.2)	306 (65.2)	270 (58.3)
University education, n (%)	1166 (65.1)	605 (70.5)	269 (57.4)	292 (63.1)
Employed, n (%)	1651 (92.2)	803 (93.6)	434 (92.5)	414 (89.4)
Psychiatric comorbidities, n (%)	595 (33.2)	280 (32.6)	173 (36.9)	142 (30.7)
Family history of psychopathology, n (%)	1218 (68.0)	597 (69.6)	301 (64.2)	320 (69.1)
Prior psychiatric diagnosis, n (%)	1091 (60.9)	553 (64.5)	286 (61.0)	252 (54.4)
Prior psychotropic medication, n (%)	1090 (60.9)	569 (66.3)	286 (61.0)	235 (50.8)

a Clinically meaningful improvement is defined as meeting the symptom reduction criterion (≥40% for depression and panic disorder and ≥30% for social anxiety disorder), the remission criterion (Montgomery-Åsberg Depression Rating Scale, Self-Rating Version [MADRS-S≤10] for depression, Panic Disorder Severity Scale, Self-Report [PDSS-SR≤7] for panic disorder, and Liebowitz Social Anxiety Scale, Self-Report [LSAS-SR≤35] for social anxiety disorder), or both.

b MADRS-S, PDSS-SR, and LSAS-SR scores were linearly rescaled to a 0 to 100 range.

The training set included patients treated from 2008 to 2017, whereas the temporal holdout test set included the most recently treated patients from 2017 to 2019. Across disorders, the training set contained a greater percentage of improved patients than the test set (63.7% vs 56.7%), and both pretreatment and posttreatment scores were higher in the test set, suggesting a temporal shift toward greater severity.

### Phenotypic Models

In nested CV, models with elastic net regularization outperformed those using internal variable selection across all methods except RF, where the omission of redundant predictors may have negatively affected tree splits by reducing ensemble diversity. We therefore proceeded with elastic net-regularized LR, XGBoost, and SVM and a nonregularized RF.

After assessing the calibration of the final models retrained on the entire training set, all models were recalibrated using a 5-fold CV. The recalibration method was selected based on the visual inspection of training set out-of-fold reliability diagrams: Platt scaling (sigmoid) was used when miscalibration showed relatively smooth deviations (benchmark model, LR, and SVM), and isotonic regression was used when deviations were less regular (RF, XGBoost, and both ensembles). The recalibrated models were then evaluated once in the temporally separated holdout test set. Consistent with the lower improvement rate observed in the holdout test set, all models showed some overprediction of the positive class, as reflected by mean predicted probabilities above the observed event rate and negative calibration-in-the-large values. Calibration metrics and curves are provided in Multimedia Appendix 7.

**Figure 2.**
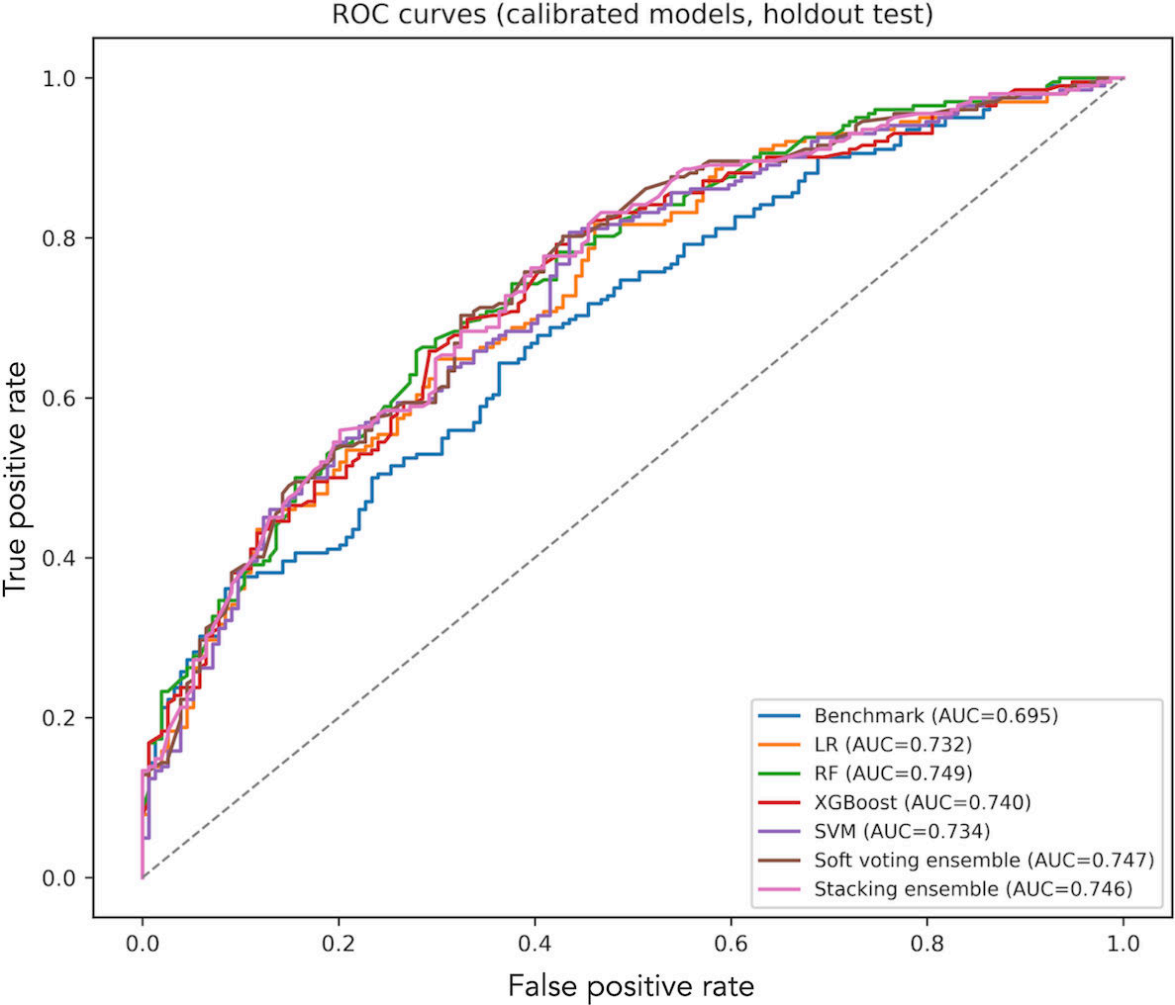
Area under the receiver operating characteristic (ROC) curve (AUC) in the holdout test set (n=356). LR: logistic regression; RF: random forest; SVM: support vector machine; XGBoost: extreme gradient boosting.

Nested CV and holdout test set performance are shown in Table 2, and receiver operating characteristic curves are presented in Figure 2. The benchmark model yielded the lowest performance (AUC_test_ 0.695, 95% CI 0.637-0.748). All full phenotypic models showed comparable performance with overlapping CIs, ranging from AUC_test_ 0.732 (95% CI 0.679-0.784) for LR to 0.749 (95% CI 0.698-0.797) for RF, followed closely by both ensembles. All 21 pairwise DeLong comparisons among the 7 models showed broadly similar performance, except for the benchmark model, which had lower discrimination than RF (*P*=.04), soft voting (*P*=.02), and stacking ensemble (*P*=.03; Multimedia Appendix 8). For threshold-dependent metrics, model-specific decision thresholds were selected on the training set using 10-fold out-of-fold predicted probabilities to maximize the Youden’s J index and were then applied unchanged to the holdout test set. Confusion matrices are shown in Multimedia Appendix 9. Secondary metrics (*F*_1_-score, log loss, and Matthews correlation coefficient) and per-class operating characteristics (sensitivity, specificity, and positive and negative predictive values) are provided in Multimedia Appendix 10.

**Table 2.** Performance of phenotypic models predicting clinically meaningful improvement in internet-delivered cognitive behavioral therapy (ICBT).

Model	AUC^a^ (95% CI)
Nested CV^b^^,c^	
Benchmark	0.676 (0.640-0.713)
Logistic regression	0.701 (0.663-0.740)
Random forest	0.690 (0.656-0.724)
XGBoost^d^	0.692 (0.661-0.724)
SVM^e^	0.700 (0.662-0.738)
Soft-voting ensemble	0.703 (0.665-0.741)
Stacking ensemble	0.702 (0.663-0.740)
Holdout test set^f^	
Benchmark	0.695 (0.637-0.748)
Logistic regression	0.732 (0.679-0.784)
Random forest	0.749 (0.698-0.797)
XGBoost	0.740 (0.689-0.790)
SVM	0.734 (0.680-0.784)
Soft-voting ensemble	0.747 (0.695-0.796)
Stacking ensemble	0.746 (0.694-0.795)

a AUC: area under the receiver operating characteristic curve.

b CV: cross-validation.

c Nested CV performance is reported with 95% CI to account for multiple‐imputation uncertainty (the Rubin’s rules).

d XGBoost: extreme gradient boosting.

e SVM: support vector machine.

f Holdout test set performance is reported with 95% bootstrap CI to quantify sampling variability.

### Sensitivity Analyses

#### Complete-Case Analysis

Complete-case nested CV yielded near-identical results to imputed models (Multimedia Appendix 11), indicating that MI with embedded variable selection is a preferable approach that preserves sample size, thus avoiding the loss of hundreds of otherwise informative observations, without degrading performance [28].

#### Expanded Phenotypic-Only Sample Without the Exclusion of Rows With Missing Genetic Data

In the larger phenotypic-only sample that did not exclude patients with missing genetic data, outcome prevalence was highly similar compared to the main analytic sample (training set: 63.8%, holdout test set: 57.8% vs 63.7% and 56.7%, respectively), suggesting limited outcome-related selection due to genetic data availability. Model performance was also nearly unchanged (AUC_test_ ranging from 0.707 to 0.762 compared with 0.695 to 0.749 in the primary analysis). Other metrics showed a similarly stable pattern, and log loss was slightly lower across all models, suggesting a slight improvement in probabilistic prediction in the larger sample. Overall, the sensitivity analysis supported the robustness of the main findings. All performance metrics and calibration curves for the expanded sample are provided in Multimedia Appendix 12.

#### Symptom Reduction and Remission Outcomes

Because a subset of patients met only one constituent component of the composite outcome (Table 1), we conducted a post hoc sensitivity analysis to examine model behavior with each component as a separate outcome. To isolate the effect of the outcome operationalization, we held the model fixed by refitting the soft-voting ensemble (the best-performing model in nested CV) to the dichotomized symptom reduction and remission outcomes, using the identical training and validation pipeline and temporal split as in the main analysis. Holdout test set performance metrics are presented in Multimedia Appendix 13. Remission showed higher discrimination (AUC_test_ 0.828, 95% CI 0.783-0.870) than symptom reduction (AUC_test_ 0.685, 95% CI 0.631-0.741), compared with the intermediate performance for the composite outcome (AUC_test_ 0.747, 95% CI 0.695-0.796). This difference is consistent with how the 2 criteria are conceptualized: remission is defined as meeting an absolute posttreatment score threshold that is strongly affected by baseline severity, whereas symptom reduction measures relative change during treatment, representing a more demanding prediction target. The 2 criteria thus behaved as related but distinct outcomes, consistent with their partial discordance and with the rationale for a composite that acknowledges either pathway as a favorable clinically relevant end state.

### Genetic Models

Genetic models were trained on the full PGS set (615 scores), the preselected reduced PGS set (293 scores), and the psychiatric disorder subset (15 scores). The reduced PGS set performed best across algorithms and was used thereafter. Comparing the difference in AUC (ΔAUC) between PGS-only and PGS + covariates (sex, age, and the first 20 principal components) showed no significant difference; therefore, covariates were excluded from the final models in the interest of parsimony.

Nested CV performance on the reduced multi-PGS set was close to chance for both algorithms. XGBoost performed slightly better (AUC_cv_ 0.534, 95% CI 0.516-0.552) than LASSO-penalized LR (AUC_cv_ 0.508, 95% CI 0.480-0.537) and was therefore selected for holdout evaluation, but it did not generalize to the holdout test set (AUC_test_ 0.512, 95% CI 0.452-0.573), indicating no meaningful predictive signal in this cohort (Multimedia Appendix 14).

### Final Ensemble

The best base learners selected from the 10-fold nested CV were a soft-voting ensemble trained on phenotypic data (AUC_cv_ 0.703, 95% CI 0.665-0.741) and an XGBoost model trained on multi-PGSs (AUC_cv_ 0.534, 95% CI 0.516-0.552). The LR meta-learner combining their out-of-sample predicted probabilities yielded an AUC_test_ of 0.747 (95% CI 0.694-0.797; Multimedia Appendix 15). The DeLong test (*P*=.97) provided no evidence of improved discrimination by combining genetic and phenotypic predictions over the phenotypic model alone.

### Predictor Importance

#### Phenotypic Models

**Figure 3.**
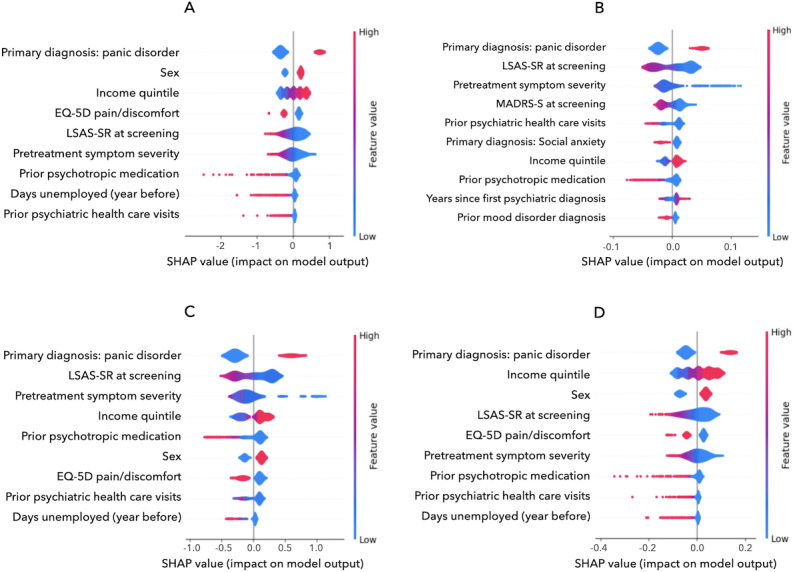
Global predictor importances for all base learners, quantified as mean absolute SHAP (Shapley Additive Explanations) values, computed on the full training set (n=1434) of the phenotypic dataset using the final refit models. (A) Logistic regression; (B) random forest; (C) extreme gradient boosting; (D) support vector machines. LSAS-SR: Liebowitz Social Anxiety Scale, Self-Report; MADRS-S: Montgomery-Åsberg Depression Rating Scale, Self-Rating Version.

SHAP (Shapley Additive Explanations) values were used for in-depth interpretation of predictor importances (Figure 3). Normalized SHAP importance percentages are reported in Multimedia Appendix 16. High agreement in the most important variables independently selected by different algorithms suggests genuinely strong predictive signals, with a PD diagnosis ranked highest. This finding reflects the higher observed treatment response rate among patients with PD (380/469, 81.0% in the total sample) compared with depression (495/858, 57.7%) and social anxiety (240/463, 51.8%).

#### Genetic Models

**Figure 4.**
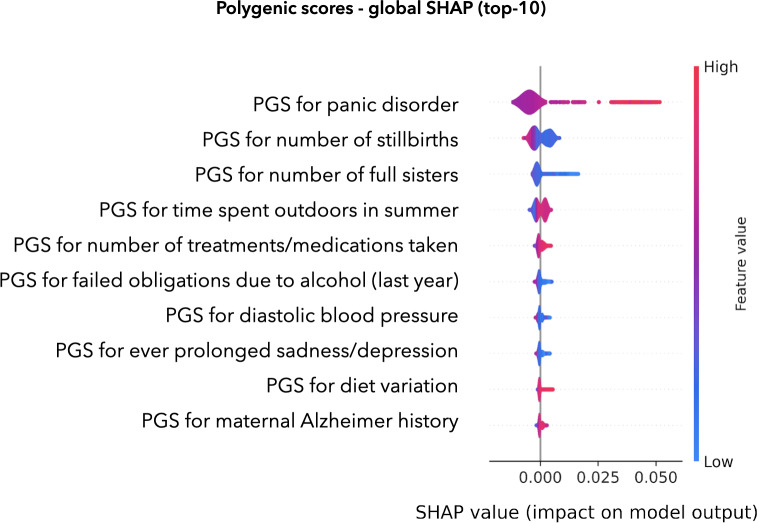
Global predictor importances for XGBoost (extreme gradient boosting; top-10 polygenic scores [PGSs]), quantified as mean absolute SHAP (Shapley Additive Explanations) values and computed on the full training set (n=1434) of the genetic dataset using the final refit model.

Exploratory bivariate analyses between the 293 PGSs and clinically meaningful improvement identified 12 associations significant at the unadjusted *α*=.05 level (Multimedia Appendix 17). Only the PD PGS remained significant under the Benjamini-Hochberg false discovery rate (FDR) at 5% (odds ratio 1.25, 95% CI 1.12-1.38, FDR-adjusted *P*=.01) with a McFadden’s pseudo-*R^2^* value of 0.008, which is in line with the PD diagnosis being the most important predictor in the phenotypic model. SHAP values of the top-10 PGSs are displayed in Figure 4.

### Secondary Analysis

Performance metrics of the regressors predicting the continuous posttreatment score on the 0 to 100 outcome scale are presented in Multimedia Appendix 18. All algorithms showed comparable performance; the soft-voting ensemble achieved the lowest RMSE_test_ of 14.27 (95% CI 12.79-15.81), MAE_test_ of 10.88 (95% CI 9.92-11.91), and *R*^2^ of 0.38 (95% CI 0.30-0.46). All algorithms yielded RMSE_test_ values between 14.27 and 14.52, each below the test set SD (20.1), indicating consistently reduced error relative to a mean-only baseline.

## Discussion

### Summary of Findings

We incorporated multimodal data to predict clinically meaningful improvement following ICBT for depression and anxiety disorders. Briefly, we trained LR, SVM, RF, XGBoost, soft voting, and stacking ensemble models on pretreatment clinical, sociodemographic, and genetic predictors from a large routine-care cohort (N=1790), using MI, elastic net variable selection, nested CV, and a temporal holdout test set. All full phenotypic models achieved moderate predictive performance, and consistent with prior results [28,41], no algorithm was clearly superior. RF and both ensembles surpassed outcome predictions made by therapists at the same clinic [27] and showed higher discrimination than the benchmark model (unadjusted DeLong tests: *P*=.04, *P*=.02, and *P*=.03, respectively). The multi-PGS model showed no predictive power (AUC_test_ 0.512, 95% CI 0.452-0.573), and the logistic meta-learner ensemble of the best phenotypic and multi-PGS models provided no evidence of a meaningful gain from PGS (*P*=.97). The holdout performance of all full phenotypic models (AUC _test_ 0.732‐0.749) compares favorably to other adequately powered and methodologically sound studies that predict CBT outcomes for depression and anxiety using baseline data only (AUCs 0.57‐0.71) [11,64-66]. Our results are thus promising and provide a strong foundation for a future prospective trial to ascertain the model’s real-world utility in the corresponding ICBT context.

### Predictors of Clinically Meaningful Improvement After ICBT

Most high-value predictors were those collected during clinical screening, highlighting the utility of easily obtainable data. The screening-based benchmark nevertheless showed lower discrimination than several full phenotypic models, although this comparison cannot fully isolate the contribution of register-based predictors from differences in modeling procedures. Further research is thus needed to determine whether self-reported equivalents of informative register-based predictors (eg, prior psychopathology, medication use, income, and unemployment) can serve as suitable proxies in settings where register linkage is not feasible. PD diagnosis was the strongest phenotypic predictor, reflecting its high response rate (81%) in this transdiagnostic sample. Moreover, the PD PGS was the strongest genetic predictor and the only PGS to survive FDR correction, likely acting as a weak genetic proxy for the clinical phenotype. This finding raised the question of whether model performance was primarily driven by predicting the subgroup for whom ICBT is the most effective treatment. To investigate this, we conducted a post hoc sensitivity analysis, retraining all full phenotypic models on a dataset excluding patients with PD (n=1321; Multimedia Appendix 19). Nested CV discrimination was equivalent for LR and soft-voting ensemble; thus, LR was taken forward to the holdout evaluation on the grounds of parsimony and interpretability, where it retained moderate discrimination (AUC_test_ 0.693, 95% CI 0.628-0.755). The modest performance drop compared to models trained on the all-disorder dataset is similar to findings from analogous analyses [66] and can be partially attributed to the smaller sample size. This result indicates that model performance was not solely driven by the PD subgroup. To isolate the predictive signal beyond diagnosis more directly, we additionally compared the full phenotypic models with a diagnosis-only model (diagnosis as the sole predictor) in the holdout test set. The full phenotypic models discriminated substantially better than the diagnosis-only model (AUC_test_ 0.625, 95% CI 0.569-0.679, DeLong *P*<.001), indicating that they captured individual-level predictive signal beyond between-disorder differences in ICBT outcomes. In practice, this suggests that the models may be most useful as an intake-stage risk stratification tool combining diagnosis with other baseline predictors.

### Role of PGSs for Prediction

PGSs did not add predictive value in the present cohort and the modeling framework. However, these results should not be interpreted as definitive evidence that genetic data are generally uninformative for psychotherapy outcome prediction. Rather, several characteristics of the current setup likely confined the detectable genetic signal. The multi-PGS framework we adopted, inspired by promising results from Albiñana et al [42], did not prove effective in the present setup, possibly due to several important differences between the studies: the authors trained their models on a much larger cohort (n>100,000), used them to predict psychiatric diagnoses with high established heritability, and included behavioral PGSs that are strongly genetically correlated with those diagnoses. Another important consideration is that our sample contains only mild-to-moderate cases with a rather high level of everyday functioning for whom ICBT is an appropriate treatment modality, whereas more severe patients are referred to specialized outpatient care. This possibly implies less enriched phenotypes and consequently, a weaker genetic signal due to an established correlation between genetic burden and disorder severity [67,68]. The complexity and polygenicity of the studied phenomenon may also play a role in the challenge to capture its genetic underpinnings. Psychotherapeutic treatment outcome is a composite, multifactorial phenotype in which genetic effects are propagated through multiple mediators related to personality traits, learning, executive function, cognition, and other factors, likely translating into diffuse associations of small effect sizes. Finally, treatment outcomes are strongly affected by environmental factors, such as adherence, homework completion, alliance, therapist effects, and idiosyncratic life events, that affect the course of therapy and cannot be accounted for in baseline predictions. Consistent with this, previous work in the same clinical setting has shown that models incorporating predictors collected during ICBT achieve substantially higher predictive accuracy [41].

### Clinical Implications and Future Directions

In clinical practice, the present models could potentially be used for risk stratification at intake by helping to identify patients at elevated risk of nonimprovement who may benefit from additional clinical attention. Plausible downstream actions may include closer monitoring, intensified therapist support, and blended or face-to-face CBT, with the ultimate aspiration to match patients with the most suitable treatment modalities. Such adaptive treatment strategies are particularly relevant in services where ICBT is delivered at scale and clinical resources need to be allocated efficiently. Importantly, while the present models prognostically predict the probability of improvement following ICBT, they do not establish that a patient predicted not to improve would benefit more from a different intervention. Matching patients to alternative treatments is therefore a future possibility that would require prospective evaluation.

A particular advantage of baseline prediction is that it may support the matching of patients to an appropriate level of treatment intensity before treatment initiation. This could help mitigate a drawback of stepped-care approaches informed by within-treatment prediction, in which a patient first undergoes a standard intervention. If this initial treatment format is not beneficial, it may prolong patient suffering, use health care resources inefficiently, and lead to patient dissatisfaction, disengagement, and delayed or reduced future help-seeking.

Temporal validation provides a useful first test of forward-looking performance within the same clinical setting, as the holdout test set consisted of more recently treated patients with higher symptom severity and a higher proportion of nonimprovement than the earlier patients. This severity drift is consistent with changes in referral and admission patterns: as ICBT became more established as an effective treatment modality for common mental health disorders, the clinic accepted a broader and more severe patient population. The models retained moderate performance in this later and more severe sample, supporting their potential relevance for near-future patients in the same clinic. Nevertheless, calibration results indicated some overall overprediction of improvement in the holdout set, consistent with the observed temporal shift in outcome prevalence. Prospective deployment should therefore include calibration monitoring and model updating to reflect current outcome prevalence.

Importantly, statistical prediction is not equivalent to clinical utility. Although the models showed moderate discrimination, their contribution to improved clinical decisions and patient outcomes will require prospective evaluation. The planning of a validation trial in new patients at the same clinic is currently underway, which will test the feasibility of baseline risk prediction under contemporary clinical conditions. Conditional on successful validation, as the next step, a prospective decision-support study will evaluate the impact of early risk stratification and model-informed care on treatment planning and patient outcomes. Finally, external validation in independent ICBT settings in other geographic locations will be required to assess model portability.

For the prospective validation trial, the soft-voting ensemble would be the primary candidate model because it achieved the highest discrimination in nested CV, while showing statistically indistinguishable holdout performance from other full phenotypic models. Before the evaluation in new incoming patients, the model will be retrained on the full available retrospective dataset.

### Strengths and Limitations

This study has several methodological strengths. First, we used a real-world clinical cohort with high-quality data collection procedures and leveraged national register linkages with excellent coverage. This allowed for the integration of multimodal data (clinical, sociodemographic, and genetic) to build a uniquely comprehensive set of predictors. The PGSs were constructed using large GWAS discovery sets, mitigating the risk that relevant SNPs go undetected in underpowered GWAS. Our models were built on a sufficiently large sample that meets criteria proposed in the literature [69-71] and exceeds the sample sizes of most prior studies [28-31]. We adhered to published guidelines on best practices of predictive model development and reporting [28,33,34]. To derive unbiased performance estimates, we used a nested CV procedure, strictly separating hyperparameter tuning from performance evaluation, and robustly handled missing data using MI nested within the CV folds. Finally, performance was validated using a temporally separated test set, providing a robust real-world estimate of generalizability to future patients.

There are several limitations relevant to the interpretation of this study. First, while efforts were made to ensure model generalizability, the current sample is somewhat clinically and sociodemographically skewed, which may hinder portability to other settings. Specifically, the ICBT clinic from which the sample was drawn is aimed at patients with mild-to-moderate symptom severity, the cohort is on average more educated than the general patient population, may have relatively higher everyday functioning, and be more motivated to undergo treatment because the majority are self-referred. These selection and self-selection biases should be considered when validating the models in new contexts. Moreover, while temporal validation evaluates robustness against data drift, external validation in independent samples will be required to ensure model portability. Second, disorder pooling introduces certain heterogeneity. However, our exploratory comparison showed that combined-data models outperformed single-disorder models, corroborating prior reports [41,72]. This suggests that the added heterogeneity may improve generalizability and mitigate overfitting, in addition to reducing maintenance costs. An additional consideration pertaining to disorder pooling is different symptom reduction thresholds used for operationalization of clinically meaningful improvement (30% for SAD and 40% for depression and PD). Symptom reduction thresholds were defined using disorder-specific criteria suggested by the literature and reflect differences in meaningful symptom reduction across instruments [48-50]. This asymmetry can in principle influence behavior of the transdiagnostic model. However, it is unlikely to account for the main diagnosis-related signal in the present pooled model because SAD still showed the lowest improvement rate and was not a prominent predictor. Instead, the lower relative change threshold for social anxiety may have attenuated between-disorder differences by making disorder subgroups more comparable. Third, the exclusion of observations with missing outcome (~20%) may introduce bias if missingness is not completely random. However, outcome imputation was not feasible as it would cause data leakage during resampling through the necessary combination of predictors with the outcome. Fourth, the reliable quantification of clinically meaningful improvement is challenging, with potential for measurement error. In the absence of objective biomarkers, improvement is a latent construct that cannot be measured directly and is thus operationalized via observable proxies (eg, psychometric scales), which are susceptible to subjectivity and stochastic noise. Construct validity is further compromised by outcome dichotomization. Finally, while substantial for a phenotypic model, the current sample is likely underpowered for a multi-PGS model. Furthermore, an inherent limitation of PGSs is that they are constructed from GWAS of common genetic variants, conferring a risk of missing potentially relevant rare variants. Importantly, the field still lacks a specific PGS for treatment outcomes.

### Conclusions

The present baseline predictive models of clinically meaningful improvement after ICBT for depression and anxiety disorders achieved moderate performance. Models incorporating register data generally showed higher discrimination than the benchmark model based on screening data, whereas PGSs added no predictive gain in this cohort and modeling setup. Our models may facilitate early identification of patients at risk of not achieving clinically meaningful improvement following ICBT, thereby informing more targeted clinical decision-making. Next steps include a prospective validation and impact evaluation in new patients at the same facility with subsequent external validation to assess the benefit of model-informed personalized care in other settings.

## Supplementary material

10.2196/100162 Multimedia Appendix 1 Changes to the preregistered protocol.

10.2196/100162 Multimedia Appendix 2 Phenotypic predictors.

10.2196/100162 Multimedia Appendix 3 Polygenic scores for psychiatric disorders.

10.2196/100162 Multimedia Appendix 4 Near-zero variance variables.

10.2196/100162 Multimedia Appendix 5 Hyperparameter tuning.

10.2196/100162 Multimedia Appendix 6 Descriptive statistics.

10.2196/100162 Multimedia Appendix 7 Calibration metrics and curves.

10.2196/100162 Multimedia Appendix 8 Pairwise DeLong tests.

10.2196/100162 Multimedia Appendix 9 Confusion matrices.

10.2196/100162 Multimedia Appendix 10 Secondary metrics.

10.2196/100162 Multimedia Appendix 11 Complete-case analysis.

10.2196/100162 Multimedia Appendix 12 Sensitivity analysis (including rows with missing genetic data).

10.2196/100162 Multimedia Appendix 13 Sensitivity analysis (symptom reduction and remission).

10.2196/100162 Multimedia Appendix 14 Performance of genetic models.

10.2196/100162 Multimedia Appendix 15 Performance of meta-ensemble.

10.2196/100162 Multimedia Appendix 16 SHAP (Shapley Additive Explanations) values.

10.2196/100162 Multimedia Appendix 17 Polygenic score associations.

10.2196/100162 Multimedia Appendix 18 Secondary analysis (continuous outcome).

10.2196/100162 Multimedia Appendix 19 Sensitivity analysis (no panic disorder).

## Abbreviations

AUC: area under the receiver operating characteristic curve
CBT: cognitive behavioral therapy
CV: cross-validation
FDR: false discovery rate
GWAS: genome-wide association studies
ICBT: internet-delivered cognitive behavioral therapy
LASSO: least absolute shrinkage and selection operator
LISA: Longitudinal Integrated Database for Health Insurance and Labor Market Studies
LR: logistic regression
LSAS-SR: Liebowitz Social Anxiety Scale, Self-Report
MADRS-S: Montgomery-Åsberg Depression Rating Scale, Self-Rating Version
MAE: mean absolute error
MI: multiple imputation
ML: machine learning
PD: panic disorder
PDSS-SR: Panic Disorder Severity Scale, Self-Report
PGS: polygenic score
PROBAST: Prediction Model Risk of Bias Assessment Tool
RF: random forest
RMSE: root mean-squared error
SAD: social anxiety disorder
SHAP: Shapley Additive Explanations
SibeR: The Swedish National Quality Register for internet-based psychological treatment
SNP: single nucleotide polymorphism
SVM: support vector machine
TRIPOD+AI: Transparent Reporting of a Multivariable Prediction Model for Individual Prognosis or Diagnosis+AI
XGBoost: extreme gradient boosting
