# Supplementary material for: Prediction of Clinically Meaningful Improvement After Internet-Delivered Cognitive Behavioral Therapy for Depression and Anxiety Disorders: Machine Learning–Based Predictive Model Development and Temporal Validation Study
Source: J Med Internet Res. 2026 Aug 7;28:e100162. doi: 10.2196/100162 (PMC13451060; doi:10.2196/100162)
Supplement: Supplementary file 1 [file jmir-v28-e100162-s001.docx]

**Changes to the pre-registered protocol [1]**

1. In addition to four individual models, we also combined them into two ensemble models.
2. As a sensitivity analysis, to appraise the impact of multiple imputation, all the models were also trained on the complete case dataset.
3. Construction of PGS for symptom change was not possible due to lack of a precise SNP-based heritability estimate.
4. We did not perform a sensitivity analysis on the dataset with imputed missing outcome values in the training set as the imputation procedure would require inclusion of predictors, thus making complete separation impossible and subsequently causing data leakage.
5. Since the preliminary results of the externally developed SNP-level predictive model were not promising, we transitioned to an alternative approach and followed the multi-PGS framework proposed by Albiñana et al. [2].
6. As listed in the pre-registered protocol, we did evaluate the selective missing indicator method (SMIM) [3] but the results were too poor to warrant a mention in the paper. A brief description of the procedure follows:

While adding a binary missing indicator for each partially observed predictor, adding many uninformative variables may introduce noise and lead to overfitting. SMIM tackles this problem by analyzing the relationship of each missing indicator with the response variable via t-test or Chi2-test for categorical and continuous variables, respectively, and applying the Benjamini-Hochberg procedure to control the false discovery rate, thus retaining only informative missing indicators. A total of 22 predictors had missing values resulting in 22 binary missing indicators whose predictiveness for outcome was assessed. Only one missing indicator passed the test and was included in the predictor set. Inclusion of this missing indicator did not boost predictive performance and was thus omitted from further analyses to reduce complexity.

***References***

1. OSF Registries osf.io/5gucx; https://osf.io/5gucx
2. Albiñana C, Zhu Z, Schork AJ, et al. Multi-PGS enhances polygenic prediction by combining 937 polygenic scores. Nat Commun. 2023;14(1):4702. Published 2023 Aug 5. doi:10.1038/s41467-023-40330-w
3. Van Ness M, Bosschieter TM, Halpin-Gregorio R, Udell M. The missing indicator method: from low to high dimensions. In: Proceedings of the 29th ACM SIGKDD Conference on Knowledge Discovery and Data Mining. Association for Computing Machinery; 2023:5004-5015. doi:10.1145/3580305.3599911
