# Supplementary material for: Prediction of Clinically Meaningful Improvement After Internet-Delivered Cognitive Behavioral Therapy for Depression and Anxiety Disorders: Machine Learning–Based Predictive Model Development and Temporal Validation Study
Source: J Med Internet Res. 2026 Aug 7;28:e100162. doi: 10.2196/100162 (PMC13451060; doi:10.2196/100162)
Supplement: Supplementary file 2 [file jmir-v28-e100162-s002.docx]

**Full list of predictors used in the phenotypic model**

| **Data source** | **Predictor** | **Definition** | **Missingness in full dataset (n=2668)** | **Comment** |
| --- | --- | --- | --- | --- |
| IPSY | Primary diagnosis: **Depression** | Patient is undergoing ICBT treatment for depression: Yes / No | 0 |  |
| IPSY | Primary diagnosis: **Panic disorder** | Patient is undergoing ICBT treatment for panic disorder: Yes / No | 0 |  |
| IPSY | Primary diagnosis: **Social anxiety** | Patient is undergoing ICBT treatment for social anxiety: Yes / No | 0 |  |
| IPSY | Pre-treatment symptom severity | MADRS-S score for patients with MDD, PDSS-SR for PD, and LSAS-SR for SAD, taken before treatment start. Respective scales harmonized to a 0-100 scale and pooled in a single variable. | 1 | For 2 patients with missing values, used a score for a respective scale collected at screening |
| Swedish personal number | Age | Number of years | 0 |  |
| Swedish personal number | Sex | Biological sex (Male/Female) | 0 | 1 patient had a missing value so the sex value was taken from NPR and verified with genetic sex |
| IPSY | Time of year of treatment start | Summer (starting in week covering April 15 and ending in week covering October 15) / Winter | 0 |  |
| IPSY & LISA | Marital status | Married (officially married or de facto married, i.e., in a committed relationship (‘married/living together/living apart together’)) / Unmarried (Single, Separated, and Widowed) | 0 | By default, used self-reported at IPSY due to better construct validity. For 6 patients with missing values, used register-based, derived from LISA. |
| IPSY & LISA | Parental status | Children / No children | 0 | By default, used self-reported at IPSY due to better construct validity. For 6 patients with missing values, used register-based, derived from LISA. |
| IPSY & LISA | Educational attainment | University (Unfinished university and Finished university) / No university (<7 years, 7-9 years, Unfinished gymnasium, Vocational school, Gymnasium) | 0 | By default, used self-reported at IPSY. For 6 patients with missing values, used register-based, derived from LISA. |
| IPSY & LISA | Psychiatric comorbidities | Comorbidity (presence of any of the 17 listed psychiatric disorders) / No comorbidity | 0 | By default, used self-reported at IPSY, but for 98 patients with missing values, use register-based comorbidity (presence of a psychiatric diagnosis in NPR or VAL, other than primary diagnosis, before treatment) |
| IPSY | Family history of psychopathology | Family history (presence of any of the 37 listed psychiatric disorders among close relatives) / No family history | 101 |  |
| IPSY | Insomnia Severity Index score | Insomnia (> 14) / No insomnia (<=14) | 86 | For 1 patient with a missing value, used a value from the screening question Other comorbid diagnoses: “Insomnia” |
| IPSY | Alcohol Use Disorders Identification Test score | Score | 100 |  |
| IPSY | Drug Use Disorders Identification Test score | Score | 7 |  |
| IPSY | EQ-5D test score for mobility | No difficulties / Some difficulties | 119 |  |
| IPSY | EQ-5D test score for anxiety / depression | No difficulties / Some difficulties / Significant difficulties | 119 |  |
| IPSY | EQ-5D test score for usual activities | No difficulties / Some difficulties / Significant difficulties | 119 |  |
| IPSY | EQ-5D test score for self-care | No difficulties / Some difficulties / Significant difficulties | 119 |  |
| IPSY | EQ-5D test score for pain / discomfort | No difficulties / Some difficulties / Significant difficulties | 119 |  |
| IPSY | CGI-S score | CGI-S, Clinical Global Impressions, Severity of Illness, clinician-rated. Numeric score (0-4 range in the sample). | 269 |  |
| IPSY | MADRS-S at screening | MADRS-S score, harmonized to a 0-100 scale, taken at screening by all patients | 0 | For 8 patients with missing values, used MADRS-S score taken before treatment |
| IPSY | PDSS-SR at screening | PDSS-SR score, harmonized to a 0-100 scale, taken at screening by all patients | 16 | For 43 patients with missing values, used PDSS-SR score taken before treatment |
| IPSY | LSAS-SR at screening | LSAS-SR score, harmonized to a 0-100 scale, taken at screening by all patients | 163 | For 2 patients with missing values, used LSAS-SR score taken before treatment |
| IPSY | Suicide risk | Clinician-rated suicide risk: Low / Medium | 102 | For 19 patients with missing values, used the suicide risk value from the clinician-rated MADRS |
| IPSY | Suicide attempts | Self-reported previous suicide attempts: Yes / No | 228 |  |
| IPSY & PDR | Current use of psychotropic medication | Self-reported currently taking any psychotropic medication: Yes / No | 0 | For 440 patients with missing values, used records of psychotropic drugs expedited within 30 days before treatment start in PDR |
| LISA | Immigrant status | Country of birth other than Sweden: Yes / No | 0 |  |
| LISA | Parental immigrant status | Mother’s or father’s country of birth other than Sweden: Yes / No | 200 |  |
| LISA | Single parent | Yes / No | 0 | Derived from Family status in LISA (codes 210 (single father with child <18) and 230 (single mother with child <18)). For 6 patients with missing values, derived from relevant LISA variables pertaining to number of children and family type. |
| LISA & IPSY | Employment status | Employed / Unemployed | 0 | For 6 patients with missing values, used self-reported |
| LISA | Income quintile | Annual disposable household income adjusted for family composition. Divided into quintiles. | 6 |  |
| LISA | Income trajectory | Percentage change in annual income from the year before treatment compared to the previous year: Growth (Increase of 20% or more) / Stable (Change within ±20%) / Reduction (Decrease of 20% or more) | 23 |  |
| NPR | Inpatient psychiatric care | Ever admitted to inpatient psychiatric care before treatment: Yes / No | 0 | Constructed from NPR Slutenvård (inpatient care), by filtering MVO (specialization) starting with 9 (Psychiatry) |
| NPR & VAL | Prior F1 diagnosis | Mental and behavioral disorders due to psychoactive substance use: Yes / No | 0 | Received any time before treatment. Duplicates between NPR and VAL are excluded. |
| NPR & VAL | Prior F3 diagnosis | Mood [affective] disorders ever before: Yes / No | 0 | Bipolar disorder is excluded and treated as a separate variable to avoid conflation with depressive disorders targeted by the studied treatment.  The depression diagnosis that a patient receives to start the ICBT treatment is transferred to the NPR. Hence, to not capture the diagnosis related to this very healthcare episode, all records from the month preceding treatment are excluded.  Duplicates between NPR and VAL are excluded. |
| NPR & VAL | Prior F4 diagnosis | Anxiety, dissociative, stress-related, somatoform and other nonpsychotic mental disorders ever before: Yes / No | 0 | The panic disorder / social anxiety diagnosis that a patient receives to start the ICBT treatment is transferred to the NPR. Hence, to not capture the diagnosis related to this very healthcare episode, all records from the month preceding treatment are excluded.  Duplicates between NPR and VAL are excluded. |
| NPR & VAL | Prior F5 diagnosis | Behavioral syndromes associated with physiological disturbances and physical factors ever before: Yes / No | 0 | Received any time before treatment.  Duplicates between NPR and VAL are excluded. |
| NPR & VAL | Prior F6 diagnosis | Disorders of adult personality and behavior ever before: Yes / No | 0 | Received any time before treatment.  Duplicates between NPR and VAL are excluded. |
| NPR & VAL | ADHD diagnosis | Attention deficit hyperactivity disorder diagnosis: Yes / No | 0 | Due to neurodevelopmental nature and persistent lifelong manifestation, not limited to diagnoses received before ICBT treatment.  Duplicates between NPR and VAL are excluded. |
| NPR & VAL | ASD diagnosis | Autism spectrum disorder diagnosis: Yes / No | 0 | Due to neurodevelopmental nature and persistent lifelong manifestation, not limited to diagnoses received before ICBT treatment.  Duplicates between NPR and VAL are excluded. |
| NPR & VAL | Number of prior healthcare visits with a psychiatric diagnosis | Number of records of primary or outpatient care visits related to a psychiatric diagnosis | 0 | For F3 and F4 diagnoses, all records from the month preceding treatment are excluded.  Duplicates between NPR and VAL are excluded. |
| NPR & VAL | Years since the first psychiatric diagnosis | Number of years | 0 | Days since any first psychiatric diagnosis, recalculated in years.  Serves as a proxy for age of onset of psychiatric morbidity. |
| PDR | Prior use of antidepressants | Yes / No | 0 | For F3 and F4 diagnoses, all records from the month preceding treatment are excluded. |
| PDR | Prior use of anxiolytics | Yes / No | 0 | For F3 and F4 diagnoses, all records from the month preceding treatment are excluded. |
| PDR | Prior use of hypnotics and sedatives | Yes / No | 0 | For F3 and F4 diagnoses, all records from the month preceding treatment are excluded. |
| PDR | Prior use of antipsychotics | Yes / No | 0 | For F3 and F4 diagnoses, all records from the month preceding treatment are excluded. |
| PDR | Number of prior expeditions of psychotropic medication | Number of expeditions | 0 |  |
| NPR & VAL | Prior CBT | Patient has previously undergone a CBT treatment: Yes / No | 0 | Records from NPR & VAL (duplicates removed) with the medical procedure code (KVÅ – medicinska åtgärder (KMÅ)) **DU011 (Cognitive behavioral therapy)**  All records from the month preceding treatment are excluded. |
| NPR & VAL | Prior other psychotherapy | Patient has previously undergone a psychotherapeutic treatment other than CBT: Yes / No | 0 | Records from NPR & VAL (duplicates removed) with the following medical procedure codes (KVÅ – medicinska åtgärder (KMÅ)):  DU008 (Psychodynamic therapy)  DU010 (Cognitive psychotherapy)  DU021 (Dialectical behavior therapy)  DU118 (Motivational interviewing)  DU013 (Mentalization-based treatment)  DU022 (Interpersonal psychotherapy)  DU119 (Relapse prevention)  DU020 (Systematic psychological therapy)  DU009 (Other specific psychological therapy)  DU032 (Mindfulness)  DV086 (Body awareness therapy)  DU007 (Supportive therapy) |
| LISA | Receipt of student income within a year before treatment | Yes / No | 6 |  |
| LISA | Days receiving sickness benefits within a year before treatment | Number of days | 6 | Sickness/work injury benefit (sjukpenning/arbetsskadesjukpenning) is paid out by the Swedish Social Insurance Agency in case of prolonged sickness (over 2 weeks) |
| LISA | Days in unemployment within a year before treatment | Number of days | 6 |  |
| LISA | Participation in active labor market policies within a year before treatment | Yes / No | 6 | Participation in active labor market policies or in subsidized employment with the Swedish Public Employment Service |
| LISA | Parental leave within a year before treatment | Yes / No | 6 |  |
| LISA | Receipt of financial benefits within a year before treatment | Any of the following:   - Rehabilitation compensation - Sickness compensation - Unemployment benefit - Compensation for participation in subsidized employment - Sickness/work injury benefit - Social assistance - Child care allowance   Yes / No | 6 | - Rehabilitation compensation (rehabiliteringsersättning) is paid out during the sickness if an individual undergoes vocational training - Sickness compensation (sjukersättning)/activity compensation (aktivitetsersättning), previously referred to as early retirement, is compensation for individuals who are prevented from working full-time ever in the future due to serious sickness, most commonly a psychiatric disorder - Unemployment benefit (arbetslöshetsersättning) applies to individuals who are currently unemployed but have worked previously - Compensation for participation in subsidized employment with the Swedish Public Employment Service (åtgärdssysselsättning) - Sickness/work injury benefit (sjukpenning/arbetsskadesjukpenning) is paid out by the Swedish Social Insurance Agency in case of prolonged sickness (over 2 weeks) - Social assistance (ekonomiskt bidrag) is provided to households that experience economic hardships and require support to pay fixed expenses - Child care allowance (vårdbidrag) is financial assistance to parents with children who have special needs |

*IPSY, Internet psychiatry clinic; ICBT, internet-delivered cognitive behavioral therapy; MADRS-S, Montgomery-Åsberg Depression Rating Scale, self-rating version; MDD, Major depressive disorder; PDSS-SR, Panic Disorder Severity Scale, self-report; PD, Panic disorder; LSAS-SR, Liebowitz Social Anxiety Scale, self-report; SAD, Social anxiety disorder; NPR, National patient register; LISA, The longitudinal integrated database for health insurance and labor market studies; VAL, The Stockholm regional healthcare data warehouse (Vårdanalysdatabasen); PDR, Prescribed drug register; ADHD, attention deficit hyperactivity disorder; ASD, autism spectrum disorder; CBT, cognitive behavioral therapy*
