# Supplementary material for: Prediction of Clinically Meaningful Improvement After Internet-Delivered Cognitive Behavioral Therapy for Depression and Anxiety Disorders: Machine Learning–Based Predictive Model Development and Temporal Validation Study
Source: J Med Internet Res. 2026 Aug 7;28:e100162. doi: 10.2196/100162 (PMC13451060; doi:10.2196/100162)
Supplement: Supplementary file 3 [file jmir-v28-e100162-s003.docx]

**List of PGS for psychiatric disorders with references to respective GWAS**

1. Depression [1]
2. Anxiety disorders [2]
3. Panic disorder [3]
4. Obsessive-compulsive disorder [4]
5. Tourette’s syndrome and other tic disorders [5]
6. Post-traumatic stress disorder [6]
7. Attention deficit hyperactivity disorder [7]
8. Autism spectrum disorder [8]
9. Bipolar disorder [9]
10. Anorexia nervosa [10]
11. Schizophrenia [11]
12. Multiple substance use disorders [12]
13. Cross-disorder PGS (meta-analysis of eight psychiatric disorders: anorexia nervosa, attention deficit hyperactivity disorder, autism spectrum disorder, bipolar disorder, major depressive disorder, obsessive-compulsive disorder, schizophrenia, and Tourette’s syndrome) [13]
14. Intelligence [14]
15. Educational attainment [15]

***References***

1. Major Depressive Disorder Working Group of the Psychiatric Genomics Consortium. Electronic address: andrew.mcintosh@ed.ac.uk; Major Depressive Disorder Working Group of the Psychiatric Genomics Consortium. Trans-ancestry genome-wide study of depression identifies 697 associations implicating cell types and pharmacotherapies. Cell. 2025;188(3):640-652.e9. doi:10.1016/j.cell.2024.12.002
2. Otowa T, Hek K, Lee M, et al. Meta-analysis of genome-wide association studies of anxiety disorders. Mol Psychiatry. 2016;21(10):1485. doi:10.1038/mp.2016.11
3. Forstner AJ, Awasthi S, Wolf C, et al. Genome-wide association study of panic disorder reveals genetic overlap with neuroticism and depression. Mol Psychiatry. 2021;26(8):4179-4190. doi:10.1038/s41380-019-0590-2
4. International Obsessive Compulsive Disorder Foundation Genetics Collaborative (IOCDF-GC) and OCD Collaborative Genetics Association Studies (OCGAS). Revealing the complex genetic architecture of obsessive-compulsive disorder using meta-analysis. Mol Psychiatry. 2018;23(5):1181-1188. doi:10.1038/mp.2017.154
5. Yu D, Sul JH, Tsetsos F, et al. Interrogating the Genetic Determinants of Tourette's Syndrome and Other Tic Disorders Through Genome-Wide Association Studies. Am J Psychiatry. 2019;176(3):217-227. doi:10.1176/appi.ajp.2018.18070857
6. Nievergelt CM, Maihofer AX, Atkinson EG, et al. Genome-wide association analyses identify 95 risk loci and provide insights into the neurobiology of post-traumatic stress disorder. Nat Genet. 2024;56(5):792-808. doi:10.1038/s41588-024-01707-9
7. Demontis D, Walters GB, Athanasiadis G, et al. Genome-wide analyses of ADHD identify 27 risk loci, refine the genetic architecture and implicate several cognitive domains. Nat Genet. 2023;55(2):198-208. doi:10.1038/s41588-022-01285-8
8. Grove J, Ripke S, Als TD, et al. Identification of common genetic risk variants for autism spectrum disorder. Nat Genet. 2019;51(3):431-444. doi:10.1038/s41588-019-0344-8
9. O'Connell KS, Koromina M, van der Veen T, et al. Genomics yields biological and phenotypic insights into bipolar disorder. Nature. 2025;639(8056):968-975. doi:10.1038/s41586-024-08468-9
10. Watson HJ, Yilmaz Z, Thornton LM, et al. Genome-wide association study identifies eight risk loci and implicates metabo-psychiatric origins for anorexia nervosa. Nat Genet. 2019;51(8):1207-1214. doi:10.1038/s41588-019-0439-2
11. Trubetskoy V, Pardiñas AF, Qi T, et al. Mapping genomic loci implicates genes and synaptic biology in schizophrenia. Nature. 2022;604(7906):502-508. doi:10.1038/s41586-022-04434-5
12. Hatoum AS, Colbert SMC, Johnson EC, et al. Multivariate genome-wide association meta-analysis of over 1 million subjects identifies loci underlying multiple substance use disorders. Nat Ment Health. 2023;1(3):210-223. doi:10.1038/s44220-023-00034-y
13. Cross-Disorder Group of the Psychiatric Genomics Consortium. Electronic address: plee0@mgh.harvard.edu; Cross-Disorder Group of the Psychiatric Genomics Consortium. Genomic Relationships, Novel Loci, and Pleiotropic Mechanisms across Eight Psychiatric Disorders. Cell. 2019;179(7):1469-1482.e11. doi:10.1016/j.cell.2019.11.020
14. Savage JE, Jansen PR, Stringer S, et al. Genome-wide association meta-analysis in 269,867 individuals identifies new genetic and functional links to intelligence. Nat Genet. 2018;50(7):912-919. doi:10.1038/s41588-018-0152-6
15. Okbay A, Wu Y, Wang N, et al. Polygenic prediction of educational attainment within and between families from genome-wide association analyses in 3 million individuals. Nat Genet. 2022;54(4):437-449. doi:10.1038/s41588-022-01016-z
