# Supplementary material for: Prediction of Clinically Meaningful Improvement After Internet-Delivered Cognitive Behavioral Therapy for Depression and Anxiety Disorders: Machine Learning–Based Predictive Model Development and Temporal Validation Study
Source: J Med Internet Res. 2026 Aug 7;28:e100162. doi: 10.2196/100162 (PMC13451060; doi:10.2196/100162)
Supplement: Supplementary file 4 [file jmir-v28-e100162-s004.docx]

**Variables excluded due to near zero variance**

1. Prior F0 diagnosis (Mental disorders due to known physiological conditions)
2. Prior F2 diagnosis (Schizophrenia, schizotypal, delusional, and other non-mood psychotic disorders)
3. Prior F7 diagnosis (Intellectual Disabilities)
4. Prior F8 diagnosis (Pervasive and specific developmental disorders), excluding autism spectrum disorder
5. Prior F9 diagnosis (Behavioral and emotional disorders with onset usually occurring in childhood and adolescence), excluding ADHD
6. Prior F99 diagnosis (Unspecified mental disorder)
7. Prior F31 diagnosis (Bipolar affective disorder)
