# Supplementary material for: Prediction of Clinically Meaningful Improvement After Internet-Delivered Cognitive Behavioral Therapy for Depression and Anxiety Disorders: Machine Learning–Based Predictive Model Development and Temporal Validation Study
Source: J Med Internet Res. 2026 Aug 7;28:e100162. doi: 10.2196/100162 (PMC13451060; doi:10.2196/100162)
Supplement: Supplementary file 5 [file jmir-v28-e100162-s005.docx]

**Hyperparameter tuning**

1. For the tuned LR models, we tuned solvers (*liblinear* with L2 penalty, *lbfgs* with L2 penalty, and *saga* with elastic net penalty) and regularization strength (C values) in the range {0.1, 0.5, 1, 5, 10}.
2. For the RF grid search, we optimized number of trees (n_estimators) {100, 300, 500}, maximum tree depth (max_depth) {None, 5, 10, 20}, minimum samples to split (min_samples_split) {2, 10, 50}, minimum samples per leaf (min_samples_leaf) {1, 2, 4, 10}, and features per split (max_features) {"sqrt", "log2"}.
3. For XGBoost, Bayesian optimization was evaluated at 50 and 100 iterations. The continuous search space for Bayesian optimization comprised number of gradient boosted trees (n_estimators) [50, 1500], learning rate [0.001, 0.5], maximum tree depth (max_depth) [3, 10], minimum sum of instance weight (hessian) needed in a child (min_child_weight) [1, 10], subsample ratio of the training instance (subsample) [0.6, 1.0], and subsample ratio of columns when constructing each tree (colsample_bytree) [0.6, 1.0].
4. The SVM grid search tested the linear kernel with the C value {0.1, 1, 10} and the RBF kernel with the C value {0.1, 1, 10} plus gamma {1e-3, 1e-4}.
