# Supplementary material for: Prediction of Clinically Meaningful Improvement After Internet-Delivered Cognitive Behavioral Therapy for Depression and Anxiety Disorders: Machine Learning–Based Predictive Model Development and Temporal Validation Study
Source: J Med Internet Res. 2026 Aug 7;28:e100162. doi: 10.2196/100162 (PMC13451060; doi:10.2196/100162)
Supplement: Supplementary file 6 [file jmir-v28-e100162-s006.docx]

**Detailed descriptive statistics for training and test sample and per disorder**

| **Variable** | **Training sample** | | | | **Test sample** | | | |
| --- | --- | --- | --- | --- | --- | --- | --- | --- |
|  | **Total**  (n=1434) | **MDD**  (n=687) | **PD**  (n=376) | **SAD**  (n=371) | **Total**  (n=356) | **MDD**  (n=171) | **PD**  (n=93) | **SAD**  (n=92) |
| Pre-treatment symptom severity (harmonized), mean (SD) | 46.6 (17.5) | 51.2 (15.3) | 39.2 (18.4) | 45.6 (17.7) | 49.0 (16.4) | 53.0 (15.5) | 42.0 (16.7) | 48.8 (15.7) |
| Post-treatment symptom severity (harmonized), mean (SD) | 27.2 (18.3) | 29.8 (18.4) | 16.2 (14.6) | 33.8 (16.8) | 31.7 (20.1) | 34.1 (19.7) | 21.5 (19.9) | 37.4 (17.3) |
| Clinically meaningful improvement, *n* (%) | 913 (63.7) | 407 (59.2) | 308 (81.9) | 198 (53.4) | 202 (56.7) | 88 (51.5) | 72 (77.4) | 42 (45.7) |
| Age, mean (SD) | 36.3 (11.6) | 38.5 (11.9) | 35.0 (11.1) | 33.5 (10.6) | 35.6 (12.0) | 37.6 (11.9) | 35.3 (12.0) | 32.1 (11.5) |
| Male sex, *n* (%) | 550 (38.4) | 226 (32.9) | 161 (42.8) | 163 (43.9) | 114 (32.0) | 53 (31.0) | 31 (33.3) | 30 (32.6) |
| Married, *n* (%) | 850 (59.3) | 392 (57.1) | 243 (64.6) | 215 (58.0) | 225 (63.2) | 107 (62.6) | 63 (67.7) | 55 (59.8) |
| Children, *n* (%) | 634 (44.2) | 344 (50.1) | 167 (44.4) | 123 (33.2) | 138 (38.8) | 78 (45.6) | 36 (38.7) | 24 (26.1) |
| Single parent, *n* (%) | 79 (5.5) | 49 (7.1) | 17 (4.5) | 13 (3.5) | 14 (3.9) | 10 (5.8) | 2 (2.2) | 2 (2.2) |
| University education, *n* (%) | 941 (65.6) | 488 (71.0) | 216 (57.4) | 237 (63.9) | 225 (63.2) | 117 (68.4) | 53 (57.0) | 55 (59.8) |
| Employed, *n* (%) | 1323 (92.3) | 645 (93.9) | 347 (92.3) | 331 (89.2) | 328 (92.1) | 158 (92.4) | 87 (93.5) | 83 (90.2) |
| Annual income (SEK), mean (SD) | 241,550 (215,530) | 248,933 (200,139) | 259,626 (290,497) | 209,638 (136,366) | 259,005 (162,166) | 284,950 (165,542) | 237,135 (142,301) | 232,889 (168,761) |
| Income trajectory |  |  |  |  |  |  |  |  |
| Growth, *n* (%) | 431 (30.1) | 183 (26.6) | 133 (35.4) | 115 (31.0) | 120 (33.7) | 55 (32.2) | 30 (32.3) | 35 (38.0) |
| Stable, *n* (%) | 765 (53.3) | 383 (55.7) | 186 (49.5) | 196 (52.8) | 190 (53.4) | 98 (57.3) | 47 (50.5) | 45 (48.9) |
| Reduction, *n* (%) | 228 (15.9) | 112 (16.3) | 57 (15.2) | 59 (15.9) | 42 (11.8) | 17 (9.9) | 14 (15.1) | 11 (12.0) |
| Receipt of financial benefits  1 year before, *n* (%) | 281 (19.6) | 156 (22.7) | 74 (19.7) | 51 (13.7) | 70 (19.7) | 37 (21.6) | 19 (20.4) | 14 (15.2) |
| Receipt of student income  1 year before, *n* (%) | 310 (21.6) | 105 (15.3) | 87 (23.1) | 118 (31.8) | 102 (28.7) | 38 (22.2) | 25 (26.9) | 39 (42.2) |
| Days receiving sickness benefits  1 year before, mean (SD) | 10.2 (43.1) | 11.8 (45.2) | 11.1 (46.3) | 6.6 (35.0) | 13.5 (50.7) | 13.8 (48.9) | 12.7 (49.5) | 13.9 (55.6) |
| Days in unemployment 1 year before, mean (SD) | 8.8 (38.5) | 9.4 (38.6) | 7.7 (36.8) | 8.8 (40.2) | 7.8 (42.6) | 10.6 (51.3) | 8.4 (43.6) | 1.8 (13.3) |
| Participation in active labor market policies 1 year before, *n* (%) | 49 (3.4) | 22 (3.2) | 11 (2.9) | 16 (4.3) | 10 (2.8) | 5 (2.9) | 4 (4.3) | 1 (1.1) |
| Parental leave 1 year before, *n* (%) | 319 (22.2) | 157 (22.9) | 88 (23.4) | 74 (19.9) | 73 (20.5) | 35 (20.5) | 24 (25.8) | 14 (15.2) |
| Immigrant 1st gen, *n* (%) | 108 (7.5) | 56 (8.2) | 28 (7.4) | 24 (6.5) | 29 (8.1) | 14 (8.2) | 10 (10.8) | 5 (5.4) |
| Immigrant 2nd gen, *n* (%) | 242 (16.9) | 121 (17.6) | 61 (16.2) | 60 (16.2) | 66 (18.5) | 31 (18.1) | 22 (23.7) | 13 (14.1) |
| Psychiatric comorbidities, *n* (%) | 462 (32.2) | 219 (31.9) | 135 (35.9) | 108 (29.1) | 133 (37.4) | 61 (35.7) | 38 (40.9) | 34 (37.0) |
| Family history of psychopathology,  *n* (%) | 946 (66.0) | 467 (68.0) | 232 (61.7) | 247 (66.6) | 272 (76.4) | 130 (76.0) | 69 (74.2) | 73 (79.3) |
| Treatment start in summer, *n* (%) | 264 (18.4) | 132 (19.2) | 66 (17.6) | 66 (17.8) | 76 (21.3) | 46 (26.9) | 19 (20.4) | 11 (12.0) |
| Insomnia, *n* (%) | 363 (25.3) | 220 (32.0) | 80 (21.3) | 63 (17.0) | 108 (30.3) | 74 (43.3) | 22 (23.7) | 12 (13.0) |
| AUDIT score, mean (SD) | 5.3 (4.4) | 5.2 (4.5) | 5.2 (4.4) | 5.4 (4.1) | 4.7 (4.2) | 4.8 (4.4) | 4.4 (4.3) | 4.7 (3.7) |
| DUDIT score, mean (SD) | 0.3 (1.3) | 0.3 (1.2) | 0.3 (1.2) | 0.4 (1.6) | 0.6 (2.4) | 0.7 (2.7) | 0.7 (2.2) | 0.5 (2.0) |
| EQ-5D Mobility |  |  |  |  |  |  |  |  |
| Some difficulties, *n* (%) | 55 (3.8) | 38 (5.5) | 14 (3.7) | 3 (0.8) | 16 (4.5) | 7 (4.1) | 6 (6.5) | 3 (3.3) |
| EQ-5D Anxiety/Depression |  |  |  |  |  |  |  |  |
| Some difficulties, *n* (%) | 918 (64.0) | 407 (59.2) | 274 (72.9) | 237 (63.9) | 233 (65.4) | 92 (53.8) | 73 (78.5) | 68 (73.9) |
| Significant difficulties, *n* (%) | 331 (23.1) | 266 (38.7) | 42 (11.2) | 23 (6.2) | 98 (27.5) | 79 (46.2) | 10 (10.8) | 9 (9.8) |
| EQ-5D Self-care |  |  |  |  |  |  |  |  |
| Some difficulties, *n* (%) | 12 (0.8) | 9 (1.3) | 3 (0.8) | 0 | 2 (0.6) | 2 (1.2) | 0 | 0 |
| Significant difficulties, *n* (%) | 1 (0.1) | 1 (0.1) | 0 | 0 | 1 (0.3) | 1 (0.6) | 0 | 0 |
| EQ-5D Pain/Discomfort |  |  |  |  |  |  |  |  |
| Some difficulties, *n* (%) | 513 (35.8) | 278 (40.5) | 159 (42.3) | 76 (20.5) | 134 (37.6) | 66 (38.6) | 39 (41.9) | 29 (31.5) |
| Significant difficulties, *n* (%) | 25 (1.7) | 14 (2.0) | 8 (2.1) | 3 (0.8) | 8 (2.2) | 6 (3.5) | 2 (2.2) | 0 |
| CGI-S score, mean (SD) |  |  |  |  |  |  |  |  |
| Normal, *n* (%) | 50 (3.5) | 27 (3.9) | 11 (2.9) | 12 (3.2) | 17 (4.8) | 9 (5.3) | 3 (3.2) | 5 (5.4) |
| Borderline ill, *n* (%) | 439 (30.6) | 215 (31.3) | 110 (29.3) | 114 (30.7) | 115 (32.3) | 59 (34.5) | 28 (30.1) | 28 (30.4) |
| Mildly ill, *n* (%) | 640 (44.6) | 309 (45.0) | 157 (41.8) | 174 (46.9) | 167 (46.9) | 84 (49.1) | 41 (44.1) | 42 (45.7) |
| Moderately ill, *n* (%) | 181 (12.6) | 72 (10.5) | 57 (15.2) | 52 (14.0) | 44 (12.4) | 18 (10.5) | 14 (15.1) | 12 (13.0) |
| Markedly ill, *n* (%) | 6 (0.4) | 0 | 5 (1.3) | 1 (0.3) | 3 (0.8) | 1 (0.6) | 2 (2.2) | 1 (1.1) |
| MADRS-S at screening (harmonized), mean (SD) | 47.5 (18.1) | 57.7 (13.7) | 38.4 (16.4) | 37.7 (16.7) | 49.4 (18.4) | 60.0 (13.7) | 38.5 (17.8) | 40.6 (15.5) |
| PDSS-SR at screening  (harmonized), mean (SD) | 27.9 (21.6) | 19.3 (19.9) | 46.1 (16.0) | 25.1 (18.4) | 27.5 (22.3) | 19.7 (19.7) | 46.0 (17.7) | 23.2 (20.4) |
| LSAS-SR at screening  (harmonized), mean (SD) | 33.8 (19.7) | 30.1 (17.4) | 24.8 (17.8) | 48.4 (16.8) | 35.8 (20.1) | 32.4 (19.8) | 27.8 (18.9) | 50.2 (13.7) |
| Suicide risk, *n* (%) | 112 (7.8) | 83 (12.1) | 6 (1.6) | 23 (6.2) | 68 (19.1) | 46 (26.9) | 13 (14.0) | 9 (9.8) |
| Suicide attempts, *n* (%) | 76 (5.3) | 42 (6.1) | 12 (3.2) | 22 (5.9) | 17 (4.8) | 7 (4.1) | 5 (5.4) | 5 (5.4) |
| Inpatient psychiatric care, *n* (%) | 67 (4.7) | 38 (5.5) | 15 (4.0) | 14 (3.8) | 14 (3.9) | 6 (3.5) | 5 (5.4) | 3 (3.3) |
| Prior psychiatric visits, mean (SD) | 3.6 (8.2) | 3.8 (7.0) | 3.3 (9.0) | 3.6 (9.3) | 6.4 (13.3) | 7.4 (14.9) | 6.3 (12.4) | 4.8 (10.6) |
| Years since the first psychiatric diagnosis, mean (SD) | 2.3 (3.3) | 2.5 (3.4) | 1.9 (3.1) | 2.2 (3.4) | 3.6 (4.6) | 3.6 (4.5) | 4.2 (4.8) | 3.1 (4.7) |
| Any prior CBT, *n* (%) | 70 (4.9) | 26 (3.8) | 17 (4.5) | 27 (7.3) | 67 (18.8) | 41 (24.0) | 14 (15.1) | 12 (13.0) |
| Any prior other psychotherapy, *n* (%) | 75 (5.2) | 41 (6.0) | 17 (4.5) | 17 (4.6) | 30 (8.4) | 16 (9.4) | 5 (5.4) | 9 (9.8) |
| Prior psychiatric diagnosis, *n* (%) | 854 (59.6) | 433 (63.0) | 220 (58.5) | 201 (54.2) | 237 (66.6) | 120 (70.2) | 66 (71.0) | 51 (55.4) |
| Prior F1 | 85 (5.9) | 41 (6.0) | 19 (5.1) | 25 (6.7) | 20 (5.6) | 7 (4.1) | 7 (7.5) | 6 (6.5) |
| Prior F3 (excl. BPAD) | 457 (31.9) | 319 (46.4) | 52 (13.8) | 86 (23.2) | 126 (35.4) | 81 (47.4) | 20 (21.5) | 25 (27.2) |
| Prior F4 | 605 (42.2) | 253 (36.8) | 199 (52.9) | 153 (41.2) | 196 (55.1) | 88 (51.5) | 63 (67.7) | 45 (48.9) |
| Prior F5 | 118 (8.2) | 72 (10.5) | 19 (5.1) | 27 (7.3) | 30 (8.4) | 19 (11.1) | 3 (3.2) | 8 (8.7) |
| Prior F6 | 26 (1.8) | 11 (1.6) | 8 (2.1) | 7 (1.9) | 5 (1.4) | 1 (0.6) | 2 (2.2) | 2 (2.2) |
| ASD diagnosis | 22 (1.5) | 11 (1.6) | 2 (0.5) | 9 (2.4) | 7 (2.0) | 4 (2.3) | 1 (1.1) | 2 (2.2) |
| ADHD diagnosis | 69 (4.8) | 31 (4.5) | 14 (3.7) | 24 (6.5) | 10 (2.8) | 3 (1.8) | 5 (5.4) | 2 (2.2) |
| Prior psychotropic medication dispensations, mean (SD) | 10.3 (22.3) | 12.6 (24.8) | 7.1 (14.5) | 9.0 (23.3) | 18.6 (64.3) | 19.8 (47.3) | 24.6 (105.0) | 10.4 (26.2) |
| Prior psychotropic medication, *n* (%) | 867 (60.5) | 448 (65.2) | 228 (60.6) | 191 (51.5) | 223 (62.6) | 121 (70.8) | 58 (62.4) | 44 (47.8) |
| Antidepressants | 693 (48.3) | 387 (56.3) | 148 (39.4) | 158 (42.6) | 188 (52.8) | 105 (61.4) | 45 (48.4) | 38 (41.3) |
| Anxiolytics | 536 (37.4) | 244 (35.5) | 178 (47.3) | 114 (30.7) | 131 (36.8) | 64 (37.4) | 41 (44.1) | 26 (38.3) |
| Hypnotics and sedatives | 373 (26.0) | 216 (31.4) | 76 (20.2) | 81 (21.8) | 93 (26.1) | 61 (35.7) | 17 (18.3) | 15 (16.3) |
| Antipsychotics | 52 (3.6) | 22 (3.2) | 14 (3.7) | 16 (4.3) | 14 (3.9) | 9 (5.3) | 4 (4.3) | 1 (1.1) |

*MDD, major depressive disorder; PD, panic disorder; SAD, social anxiety disorder; SEK, Swedish krona; AUDIT, Alcohol Use Disorders Identification Test; DUDIT, Drug Use Disorders Identification Test; CGI-S, Clinical Global Impressions, Severity of Illness, clinician-rated; MADRS-S, Montgomery-Åsberg Depression Rating Scale, self-rating version; PDSS-SR, Panic Disorder Severity Scale, self-report; LSAS-SR, Liebowitz Social Anxiety Scale, self-report; ASD, autism spectrum disorder; ADHD, attention deficit hyperactivity disorder*
