# Supplementary material for: Prediction of Clinically Meaningful Improvement After Internet-Delivered Cognitive Behavioral Therapy for Depression and Anxiety Disorders: Machine Learning–Based Predictive Model Development and Temporal Validation Study
Source: J Med Internet Res. 2026 Aug 7;28:e100162. doi: 10.2196/100162 (PMC13451060; doi:10.2196/100162)
Supplement: Supplementary file 7 [file jmir-v28-e100162-s007.docx]

**Calibration metrics of the recalibrated models on the holdout test set (phenotypic models)^a^**

|  | **Benchmark** | **LR** | **RF** | **XGBoost** | **SVM** | **Soft voting ensemble** | **Stacking ensemble** |
| --- | --- | --- | --- | --- | --- | --- | --- |
| Observed event rate | 0.567 | 0.567 | 0.567 | 0.567 | 0.567 | 0.567 | 0.567 |
| Mean predicted probability | 0.624 | 0.620 | 0.609 | 0.616 | 0.626 | 0.619 | 0.617 |
| Calibration-in-the-large | -0.261 | -0.250 | -0.194 | -0.227 | -0.277 | -0.248 | -0.240 |
| Calibration slope | 1.225 | 1.174 | 1.379 | 1.305 | 1.213 | 1.136 | 1.131 |
| Brier score | 0.219 | 0.211 | 0.204 | 0.208 | 0.211 | 0.207 | 0.206 |

*LR, logistic regression; RF, random forest; SVM, support vector machines*

*^a^Calibration was evaluated for the final models after retraining on the entire training set. Before single-pass evaluation on the holdout test set, each model was recalibrated by 5-fold cross-validation on the training set, using Platt scaling (sigmoid) for the benchmark model, logistic regression (LR), and support vector machines (SVM), and isotonic regression for random forest (RF), XGBoost, and soft voting and stacking ensembles. Calibration-in-the-large values below 0 indicate overall overprediction, whereas values above 0 indicate underprediction. Brier score summarizes overall probabilistic prediction error, with lower values indicating better performance.*

**Calibration curves of the recalibrated models on the holdout test set (phenotypic models)**

*β₀ denotes the calibration intercept, β₁ the calibration slope, and Br the Brier score*
