# Supplementary material for: Prediction of Clinically Meaningful Improvement After Internet-Delivered Cognitive Behavioral Therapy for Depression and Anxiety Disorders: Machine Learning–Based Predictive Model Development and Temporal Validation Study
Source: J Med Internet Res. 2026 Aug 7;28:e100162. doi: 10.2196/100162 (PMC13451060; doi:10.2196/100162)
Supplement: Supplementary file 8 [file jmir-v28-e100162-s008.docx]

**Pairwise DeLong tests comparing AUC of holdout test set for calibrated phenotypic models**

| **Model 1** | **Model 2** | **z** | ***p*-value** |
| --- | --- | --- | --- |
| Benchmark | Logistic regression | -1.403 | .161 |
| Benchmark | Random forest | -2.014 | .044* |
| Benchmark | XGBoost | -1.634 | .102 |
| Benchmark | SVM | -1.578 | .114 |
| Benchmark | Soft voting ensemble | -2.345 | .019* |
| Benchmark | Stacking ensemble | -2.246 | .025* |
| Logistic regression | RF | -0.559 | .576 |
| Logistic regression | XGBoost | -0.250 | .802 |
| Logistic regression | SVM | -0.175 | .861 |
| Logistic regression | Soft voting ensemble | -1.935 | .053 |
| Logistic regression | Stacking ensemble | -1.918 | .055 |
| Random forest | XGBoost | -0.300 | .764 |
| Random forest | SVM | -0.447 | .655 |
| Random forest | Soft voting ensemble | -0.048 | .961 |
| Random forest | Stacking ensemble | -0.075 | .940 |
| XGBoost | SVM | -0.193 | .847 |
| XGBoost | Soft voting ensemble | -0.294 | .768 |
| XGBoost | Stacking ensemble | -0.246 | .806 |
| SVM | Soft voting ensemble | -1.609 | .108 |
| SVM | Stacking ensemble | -1.449 | .147 |
| Soft voting ensemble | Stacking ensemble | -0.139 | .889 |

** P*<0.05
