# Supplementary material for: Prediction of Clinically Meaningful Improvement After Internet-Delivered Cognitive Behavioral Therapy for Depression and Anxiety Disorders: Machine Learning–Based Predictive Model Development and Temporal Validation Study
Source: J Med Internet Res. 2026 Aug 7;28:e100162. doi: 10.2196/100162 (PMC13451060; doi:10.2196/100162)
Supplement: Supplementary file 9 [file jmir-v28-e100162-s009.docx]

**Confusion matrices of the holdout test set performance (phenotypic models)^a^**

*^a^Confusion matrices at model-specific thresholds, selected on the training data and applied unchanged to the holdout test set*
