# Supplementary material for: Prediction of Clinically Meaningful Improvement After Internet-Delivered Cognitive Behavioral Therapy for Depression and Anxiety Disorders: Machine Learning–Based Predictive Model Development and Temporal Validation Study
Source: J Med Internet Res. 2026 Aug 7;28:e100162. doi: 10.2196/100162 (PMC13451060; doi:10.2196/100162)
Supplement: Supplementary file 10 [file jmir-v28-e100162-s010.docx]

**Performance of phenotypic models predicting clinically meaningful improvement post-ICBT (secondary metrics)**

|  | **Benchmark** | **LR** | **RF** | **XGBoost** | **SVM** | **Soft voting ensemble** | **Stacking ensemble** |
| --- | --- | --- | --- | --- | --- | --- | --- |
| **Nested CV^a^** |  |  |  |  |  |  |  |
| BACC | 0.621  [0.585, 0.657] | 0.645  [0.610, 0.679] | 0.625  [0.594, 0.656] | 0.646  [0.624, 0.668] | 0.640  [0.612, 0.667] | 0.637  [0.597, 0.676] | 0.604  [0.578, 0.631] |
| F1 | 0.663  [0.629, 0.697] | 0.697  [0.666, 0.729] | 0.674  [0.649, 0.699] | 0.668  [0.651, 0.685] | 0.679  [0.652, 0.705] | 0.724  [0.692, 0.757] | 0.769  [0.748, 0.791] |
| Log loss | 0.648  [0.625, 0.672] | 0.631  [0.607, 0.656] | 0.630  [0.618, 0.643] | 0.639  [0.625, 0.652] | 0.600  [0.572, 0.628] | 0.615  [0.598, 0.632] | 0.597  [0.571, 0.624] |
| MCC | 0.233  [0.164, 0.302] | 0.280  [0.213, 0.348] | 0.242  [0.183, 0.300] | 0.282  [0.239, 0.324] | 0.269  [0.215, 0.323] | 0.272  [0.193, 0.350] | 0.245  [0.185, 0.304] |
| **Holdout test set^b^** | | | | | | | |
| BACC | 0.610  [0.560, 0.658] | 0.657  [0.610, 0.706] | 0.670  [0.622, 0.717] | 0.657  [0.608, 0.705] | 0.659  [0.610, 0.709] | 0.670  [0.621, 0.718] | 0.674  [0.625, 0.723] |
| F1 | 0.548  [0.478, 0.610] | 0.638  [0.579, 0.696] | 0.665  [0.607, 0.718] | 0.659  [0.600, 0.713] | 0.681  [0.625, 0.731] | 0.693  [0.638, 0.742] | 0.693  [0.638, 0.745] |
| Log loss | 0.624  [0.588, 0.663] | 0.608  [0.567, 0.648] | 0.589  [0.555, 0.624] | 0.597  [0.561, 0.634] | 0.609  [0.569, 0.650] | 0.596  [0.556, 0.637] | 0.594  [0.554, 0.635] |
| MCC | 0.229  [0.126, 0.327] | 0.316  [0.221, 0.413] | 0.340  [0.244, 0.434] | 0.311  [0.214, 0.406] | 0.316  [0.218, 0.413] | 0.337  [0.240, 0.433] | 0.345  [0.249, 0.443] |

*ICBT, internet-delivered cognitive behavioral therapy; LR, logistic regression; RF, random forest; SVM, support vector machines; CV, cross-validation; BACC, balanced accuracy; MCC, the Matthews correlation coefficient*

*^a^Nested CV outer fold performance is reported with 95% CI to account for multiple‐imputation uncertainty (Rubin’s rules)*

*^b^Holdout test set performance is reported with 95% bootstrap CI to quantify sampling variability*

**Per-class operating characteristics at decision thresholds selected on the training set and applied to the holdout test set^a^**

|  | **Benchmark** | **LR** | **RF** | **XGBoost** | **SVM** | **Soft voting ensemble** | **Stacking ensemble** |
| --- | --- | --- | --- | --- | --- | --- | --- |
| **Threshold** | 0.679 | 0.660 | 0.614 | 0.613 | 0.630 | 0.614 | 0.626 |
| **Sensitivity** | 0.441  [0.372, 0.510] | 0.554  [0.486, 0.624] | 0.594  [0.526, 0.662] | 0.599  [0.530, 0.664] | 0.644  [0.577, 0.708] | 0.658  [0.592, 0.722] | 0.653  [0.588, 0.718] |
| **Specificity** | 0.779  [0.712, 0.843] | 0.760  [0.690, 0.825] | 0.747  [0.677, 0.815] | 0.714  [0.642, 0.783] | 0.675  [0.598, 0.749] | 0.682  [0.605, 0.755] | 0.695  [0.622, 0.767] |
| **PPV** | 0.724  [0.640, 0.803] | 0.752  [0.681, 0.821] | 0.755  [0.688, 0.817] | 0.733  [0.667, 0.798] | 0.722  [0.655, 0.787] | 0.731  [0.667, 0.793] | 0.737  [0.670, 0.800] |
| **NPV** | 0.515  [0.450, 0.579] | 0.565  [0.495, 0.633] | 0.584  [0.515, 0.654] | 0.576  [0.506, 0.644] | 0.591  [0.518, 0.665] | 0.603  [0.531, 0.678] | 0.605  [0.530, 0.676] |

*PPV, positive predictive value; NPV, negative predictive value; LR, logistic regression; RF, random forest; SVM, support vector machines*

*^a^Youden’s J threshold learned on the training set and applied unchanged to the holdout test set*
