# Supplementary material for: Prediction of Clinically Meaningful Improvement After Internet-Delivered Cognitive Behavioral Therapy for Depression and Anxiety Disorders: Machine Learning–Based Predictive Model Development and Temporal Validation Study
Source: J Med Internet Res. 2026 Aug 7;28:e100162. doi: 10.2196/100162 (PMC13451060; doi:10.2196/100162)
Supplement: Supplementary file 11 [file jmir-v28-e100162-s011.docx]

**Complete-case sensitivity analysis. Performance of phenotypic models predicting clinically meaningful improvement post-ICBT on a complete-case dataset (n=1381).**

|  | **Benchmark** | **LR** | **RF** | **XGBoost** | **SVM** | **Soft voting ensemble** | **Stacking ensemble** |
| --- | --- | --- | --- | --- | --- | --- | --- |
| **Nested CV** |  |  |  |  |  |  |  |
| AUC | 0.681 | 0.703 | 0.698 | 0.692 | 0.703 | 0.707 | 0.704 |
| BACC | 0.622 | 0.628 | 0.641 | 0.630 | 0.641 | 0.634 | 0.606 |
| F1 | 0.667 | 0.688 | 0.676 | 0.658 | 0.688 | 0.728 | 0.781 |
| Log loss | 0.641 | 0.627 | 0.619 | 0.631 | 0.591 | 0.605 | 0.588 |
| MCC | 0.234 | 0.247 | 0.270 | 0.249 | 0.270 | 0.267 | 0.258 |
| **Holdout test set^a^** |  |  |  |  |  |  |  |
| AUC | 0.683  [0.630, 0.738] | 0.733  [0.676, 0.786] | 0.746  [0.692, 0.797] | 0.739  [0.684, 0.789] | 0.727  [0.671, 0.782] | 0.742  [0.688, 0.794] | 0.742  [0.687, 0.793] |
| BACC | 0.617  [0.566, 0.673] | 0.663  [0.612, 0.719] | 0.672  [0.624, 0.724] | 0.670  [0.625, 0.719] | 0.625  [0.577, 0.673] | 0.677  [0.624, 0.728] | 0.653  [0.600, 0.701] |
| F1 | 0.634  [0.570, 0.695] | 0.665  [0.605, 0.726] | 0.651  [0.586, 0.710] | 0.645  [0.580, 0.707] | 0.742  [0.691, 0.789] | 0.706  [0.645, 0.759] | 0.757  [0.707, 0.804] |
| Log loss | 0.634  [0.593, 0.673] | 0.609  [0.573, 0.643] | 0.605  [0.576, 0.633] | 0.615  [0.584, 0.643] | 0.613  [0.565, 0.666] | 0.598  [0.569, 0.629] | 0.604  [0.562, 0.647] |
| MCC | 0.233  [0.131, 0.340] | 0.324  [0.222, 0.434] | 0.346  [0.251, 0.451] | 0.343  [0.253, 0.439] | 0.295  [0.181, 0.398] | 0.352  [0.246, 0.451] | 0.352  [0.233, 0.457] |

*ICBT, internet-delivered cognitive behavioral therapy; LR, logistic regression; RF, random forest; SVM, support vector machines; CV, cross-validation; AUC, area under the receiver operating characteristic curve; BACC, balanced accuracy; MCC, the Matthews correlation coefficient*

*^a^Holdout test set performance is reported with 95% bootstrap CI to quantify sampling variability*
