# Supplementary material for: Prediction of Clinically Meaningful Improvement After Internet-Delivered Cognitive Behavioral Therapy for Depression and Anxiety Disorders: Machine Learning–Based Predictive Model Development and Temporal Validation Study
Source: J Med Internet Res. 2026 Aug 7;28:e100162. doi: 10.2196/100162 (PMC13451060; doi:10.2196/100162)
Supplement: Supplementary file 12 [file jmir-v28-e100162-s012.docx]

**Sensitivity analysis: Performance of phenotypic models without excluding rows with missing genetic data (n=2143)**

|  | **Benchmark** | **LR** | **RF** | **XGBoost** | **SVM** | **Soft voting ensemble** | **Stacking ensemble** |
| --- | --- | --- | --- | --- | --- | --- | --- |
| **Nested CV^a^** |  |  |  |  |  |  |  |
| AUC | 0.675  [0.656, 0.695] | 0.694  [0.671, 0.717] | 0.692  [0.675, 0.709] | 0.687  [0.666, 0.708] | 0.694  [0.669, 0.719] | 0.699  [0.678, 0.721] | 0.698  [0.677, 0.719] |
| BACC | 0.624  [0.605, 0.643] | 0.638  [0.617, 0.659] | 0.630  [0.610, 0.650] | 0.631  [0.611, 0.651] | 0.636  [0.614, 0.658] | 0.638  [0.619, 0.657] | 0.637  [0.617, 0.657] |
| F1 | 0.669  [0.648, 0.690] | 0.691  [0.670, 0.713] | 0.688  [0.670, 0.706] | 0.664  [0.647, 0.681] | 0.677  [0.657, 0.696] | 0.723  [0.705, 0.740] | 0.674  [0.658, 0.690] |
| Log loss | 0.646  [0.633, 0.659] | 0.635  [0.620, 0.649] | 0.626  [0.620, 0.633] | 0.635  [0.626, 0.643] | 0.603  [0.583, 0.623] | 0.614  [0.603, 0.625] | 0.632  [0.620, 0.644] |
| MCC | 0.238  [0.204, 0.271] | 0.265  [0.226, 0.305] | 0.252  [0.213, 0.292] | 0.252  [0.212, 0.291] | 0.261  [0.217, 0.304] | 0.270  [0.234, 0.307] | 0.264  [0.225, 0.303] |
| **Holdout test set^b^** | | | | | | | |
| AUC | 0.707  [0.658, 0.753] | 0.740  [0.692, 0.786] | 0.762  [0.716, 0.804] | 0.748  [0.702, 0.793] | 0.742  [0.694, 0.787] | 0.758  [0.711, 0.802] | 0.758  [0.712, 0.802] |
| BACC | 0.641  [0.597, 0.683] | 0.666  [0.622, 0.708] | 0.667  [0.625, 0.708] | 0.663  [0.618, 0.706] | 0.663  [0.617, 0.707] | 0.670  [0.625, 0.713] | 0.667  [0.622, 0.710] |
| F1 | 0.600  [0.540, 0.653] | 0.668  [0.617, 0.715] | 0.620  [0.561, 0.674] | 0.646  [0.591, 0.697] | 0.674  [0.621, 0.720] | 0.654  [0.601, 0.704] | 0.645  [0.588, 0.695] |
| Log loss | 0.617  [0.586, 0.650] | 0.599  [0.565, 0.635] | 0.580  [0.546, 0.613] | 0.589  [0.556, 0.623] | 0.598  [0.565, 0.633] | 0.582  [0.548, 0.618] | 0.581  [0.546, 0.614] |
| MCC | 0.289  [0.200, 0.373] | 0.330  [0.241, 0.414] | 0.342  [0.259, 0.424] | 0.325  [0.236, 0.412] | 0.323  [0.232, 0.409] | 0.339  [0.249, 0.425] | 0.335  [0.246, 0.421] |

*^a^Nested CV outer fold performance is reported with 95% CI to account for multiple‐imputation uncertainty (Rubin’s rules)*

*^b^Holdout test set performance is reported with 95% bootstrap CI to quantify sampling variability*

**Absolute difference of AUC_test_ between the primary sample (n=1790) and the expanded phenotypic-only sample (n=2143)^a^**

| **Model** | **AUC_test_**  **(n=1790)** | **AUC_test_**  **(n=2143)** | **ΔAUC** |
| --- | --- | --- | --- |
| Benchmark | 0.695 [0.637, 0.748] | 0.707 [0.658, 0.753] | +0.012 |
| Logistic regression | 0.732 [0.679, 0.784] | 0.740 [0.692, 0.786] | +0.008 |
| Random forest | 0.749 [0.698, 0.797] | 0.762 [0.716, 0.804] | +0.013 |
| XGBoost | 0.740 [0.689, 0.790] | 0.748 [0.702, 0.793] | +0.008 |
| SVM | 0.734 [0.680, 0.784] | 0.742 [0.694, 0.787] | +0.008 |
| Soft voting ensemble | 0.747 [0.695, 0.796] | 0.758 [0.711, 0.802] | +0.011 |
| Stacking ensemble | 0.746 [0.694, 0.795] | 0.758 [0.712, 0.802] | +0.012 |

*^a^Differences are descriptive and were not formally tested because the analyses were conducted in partially different analytic samples. Positive values indicate higher AUC_test_ in the expanded phenotypic-only sample.*

**Per-class operating characteristics in the expanded phenotypic-only sample at decision thresholds selected on the training set and applied to the holdout test set^a^**

|  | **Benchmark** | **LR** | **RF** | **XGBoost** | **SVM** | **Soft voting ensemble** | **Stacking ensemble** |
| --- | --- | --- | --- | --- | --- | --- | --- |
| **Threshold** | 0.672 | 0.655 | 0.693 | 0.644 | 0.646 | 0.649 | 0.635 |
| **Sensitivity** | 0.494  [0.431, 0.554] | 0.599  [0.539, 0.659] | 0.506  [0.442, 0.569] | 0.559  [0.496, 0.622] | 0.615  [0.554, 0.674] | 0.567  [0.506, 0.629] | 0.551  [0.488, 0.611] |
| **Specificity** | 0.789  [0.728, 0.847] | 0.733  [0.669, 0.795] | 0.828  [0.775, 0.880] | 0.767  [0.704, 0.826] | 0.711  [0.644, 0.778] | 0.772  [0.709, 0.832] | 0.783  [0.720, 0.842] |
| **PPV** | 0.762  [0.694, 0.825] | 0.755  [0.693, 0.812] | 0.801  [0.739, 0.860] | 0.767  [0.703, 0.826] | 0.745  [0.684, 0.801] | 0.773  [0.712, 0.833] | 0.777  [0.714, 0.840] |
| **NPV** | 0.532  [0.469, 0.590] | 0.571  [0.507, 0.635] | 0.550  [0.490, 0.608] | 0.559  [0.496, 0.622] | 0.574  [0.511, 0.639] | 0.565  [0.500, 0.627] | 0.560  [0.496, 0.620] |

*PPV, positive predictive value; NPV, negative predictive value; LR, logistic regression; RF, random forest; SVM, support vector machines*

*^a^Youden’s J threshold learned on the training set and applied unchanged to the holdout test set*

**ROC curves for calibrated phenotypic models in the expanded phenotypic-only holdout test set**

**Calibration curves of the recalibrated phenotypic models in the expanded phenotypic-only holdout test set**

*β₀ denotes the calibration intercept, β₁ the calibration slope, and Br the Brier score*
