# Supplementary material for: Prediction of Clinically Meaningful Improvement After Internet-Delivered Cognitive Behavioral Therapy for Depression and Anxiety Disorders: Machine Learning–Based Predictive Model Development and Temporal Validation Study
Source: J Med Internet Res. 2026 Aug 7;28:e100162. doi: 10.2196/100162 (PMC13451060; doi:10.2196/100162)
Supplement: Supplementary file 13 [file jmir-v28-e100162-s013.docx]

**Sensitivity analysis: Prediction of Symptom reduction and Remission (holdout performance)**

|  | **Symptom reduction** | **Remission** | **Clinically meaningful improvement** |
| --- | --- | --- | --- |
| Holdout test set outcome prevalence | 0.520 | 0.435 | 0.567 |
| AUC | 0.685  [0.631, 0.741] | 0.828  [0.783, 0.870] | 0.747  [0.695, 0.796] |
| BACC | 0.641  [0.591, 0.690] | 0.752  [0.704, 0.796] | 0.670  [0.621, 0.718] |
| F1 | 0.613  [0.547, 0.673] | 0.731  [0.673, 0.782] | 0.693  [0.638, 0.742] |
| Log loss | 0.653  [0.615, 0.692] | 0.510  [0.456, 0.568] | 0.596  [0.556, 0.637] |
| MCC | 0.286  [0.185, 0.384] | 0.499  [0.405, 0.587] | 0.337  [0.240, 0.433] |
| Threshold^a^ | 0.576 | 0.406 | 0.614 |
| Brier score | 0.229  [0.213, 0.246] | 0.166  [0.146, 0.188] | 0.207  [0.190, 0.225] |
| Calibration intercept | -0.201 | -0.055 | -0.306 |
| Calibration slope | 0.874 | 1.034 | 1.136 |
| Sensitivity | 0.551  [0.478, 0.623] | 0.787  [0.720, 0.850] | 0.658  [0.592, 0.722] |
| Specificity | 0.731  [0.663, 0.796] | 0.716  [0.653, 0.777] | 0.682  [0.605, 0.755] |
| PPV | 0.689  [0.614, 0.764] | 0.682  [0.612, 0.747] | 0.731  [0.667, 0.793] |
| NPV | 0.601  [0.534, 0.668] | 0.814  [0.753, 0.869] | 0.603  [0.531, 0.678] |

*AUC, area under the receiver operating characteristic curve; BACC, balanced accuracy; MCC, the Matthews correlation coefficient; PPV, positive predictive value; NPV, negative predictive value*

*Symptom reduction is coded as 1 if reaching the thresholds for pre- to post-treatment relative change: ≥40% for depression and panic disorder and ≥30% for social anxiety disorder. Remission is coded as 1 if reaching the post-treatment score cutoffs: MADRS-S ≤10, PDSS-SR ≤7, and LSAS-SR ≤35. Clinically meaningful improvement is coded as 1 if reaching Symptom reduction, Remission, or both.*

*Holdout test set performance is reported with 95% bootstrap CI to quantify sampling variability.*

*^a^Youden’s J threshold learned on the training set and applied unchanged to the holdout test set*
