# Supplementary material for: Prediction of Clinically Meaningful Improvement After Internet-Delivered Cognitive Behavioral Therapy for Depression and Anxiety Disorders: Machine Learning–Based Predictive Model Development and Temporal Validation Study
Source: J Med Internet Res. 2026 Aug 7;28:e100162. doi: 10.2196/100162 (PMC13451060; doi:10.2196/100162)
Supplement: Supplementary file 14 [file jmir-v28-e100162-s014.docx]

**Performance of genetic models predicting clinically meaningful improvement in ICBT**

| **Model** | **AUC** | | **BACC** | **F1** | **Log Loss** | **MCC** |
| --- | --- | --- | --- | --- | --- | --- |
| **Nested CV^a^** |  |  |  |  |  |  |
| LR with LASSO | 0.508  [0.480, 0.537] | | 0.507  [0.484, 0.530] | 0.482  [0.364, 0.601] | 0.699  [0.683, 0.716] | 0.013  [-0.031, 0.057] |
| XGBoost | 0.534  [0.516, 0.552] | | 0.518  [0.498, 0.538] | 0.563  [0.499, 0.627] | 0.689  [0.686, 0.691] | 0.036  [-0.005, 0.076] |
| **Holdout test set^b^** |  | |  |  |  |  |
| XGBoost | 0.512  [0.452, 0.573] | | 0.509  [0.461, 0.558] | 0.410  [0.338, 0.479] | 0.693  [0.693, 0.694] | 0.019  [-0.085, 0.125] |

*ICBT, internet-delivered cognitive behavioral therapy; AUC, the area under the receiver operating characteristic curve; BACC, balanced accuracy; MCC, the Matthews correlation coefficient; CV, cross-validation; LR, logistic regression*

*^a^Nested CV outer fold performance is reported as mean [95% CI] across the 10 outer folds*

*^b^Holdout test set performance is reported with 95% bootstrap CI to quantify sampling variability*
