# Supplementary material for: Prediction of Clinically Meaningful Improvement After Internet-Delivered Cognitive Behavioral Therapy for Depression and Anxiety Disorders: Machine Learning–Based Predictive Model Development and Temporal Validation Study
Source: J Med Internet Res. 2026 Aug 7;28:e100162. doi: 10.2196/100162 (PMC13451060; doi:10.2196/100162)
Supplement: Supplementary file 15 [file jmir-v28-e100162-s015.docx]

**Holdout test set performance of meta-ensemble (phenotypic and genetic models) predicting clinically meaningful improvement in ICBT^a^**

| **AUC** | **BACC** | **F1** | **Log loss** | **MCC** | **Threshold^b^** | **Sensitivity** | **Specificity** | **PPV** | **NPV** |
| --- | --- | --- | --- | --- | --- | --- | --- | --- | --- |
| 0.747  [0.694, 0.797] | 0.673  [0.623, 0.722] | 0.696  [0.642, 0.746] | 0.606  [0.571, 0.643] | 0.342  [0.244, 0.439] | 0.629 | 0.663  [0.598, 0.728] | 0.682  [0.609, 0.755] | 0.732  [0.668, 0.792] | 0.607  [0.534, 0.680] |

*ICBT, internet-delivered cognitive behavioral therapy; AUC, area under the receiver operating characteristic curve; BACC, balanced accuracy; MCC, the Matthews correlation coefficient; PPV, positive predictive value; NPV, negative predictive value*

*^a^Holdout test set performance is reported with 95% bootstrap CI to quantify sampling variability*

*^b^Youden’s J threshold learned on the training set and applied unchanged to the holdout test set*
