# Supplementary material for: Prediction of Clinically Meaningful Improvement After Internet-Delivered Cognitive Behavioral Therapy for Depression and Anxiety Disorders: Machine Learning–Based Predictive Model Development and Temporal Validation Study
Source: J Med Internet Res. 2026 Aug 7;28:e100162. doi: 10.2196/100162 (PMC13451060; doi:10.2196/100162)
Supplement: Supplementary file 16 [file jmir-v28-e100162-s016.docx]

**Normalized global SHAP importances for all base learners**

| **Model** | **Predictor** | **mean(\|SHAP\|)^a^** | **SHAP (%)^b^** |
| --- | --- | --- | --- |
| Logistic regression | Primary diagnosis: Panic disorder | 0.456994 | 26.8 |
|  | Sex | 0.216262 | 12.7 |
|  | Income quintile | 0.214545 | 12.6 |
|  | EQ-5D Pain/discomfort | 0.201858 | 11.9 |
|  | LSAS-SR at screening | 0.194155 | 11.4 |
|  | Pre-treatment symptom severity | 0.18274 | 10.7 |
|  | Prior dispensations of psychotropic medication | 0.113973 | 6.7 |
|  | Days unemployed (year pre-treatment) | 0.06889 | 4.0 |
|  | Prior psychiatric healthcare visits | 0.053767 | 3.2 |
| Random forest | Primary diagnosis: Panic disorder | 0.030976 | 14.0 |
|  | LSAS-SR at screening | 0.025793 | 11.7 |
|  | Pre-treatment symptom severity | 0.020155 | 9.2 |
|  | MADRS-S at screening | 0.013955 | 6.3 |
|  | Prior psychiatric healthcare visits | 0.012341 | 5.6 |
|  | Primary diagnosis: Social anxiety | 0.010638 | 4.8 |
|  | Income quintile | 0.010539 | 4.8 |
|  | Prior dispensations of psychotropic medication | 0.009788 | 4.4 |
|  | Years since first psychiatric diagnosis | 0.007101 | 3.2 |
|  | Prior mood disorder diagnosis | 0.006584 | 3.0 |
| XGBoost | Primary diagnosis: Panic disorder | 0.375096 | 23.9 |
|  | LSAS-SR at screening | 0.23547 | 15.0 |
|  | Pre-treatment symptom severity | 0.225392 | 14.4 |
|  | Income quintile | 0.167049 | 10.6 |
|  | Prior dispensations of psychotropic medication | 0.149109 | 9.5 |
|  | Sex | 0.131955 | 8.4 |
|  | EQ-5D Pain/discomfort | 0.130008 | 8.3 |
|  | Prior psychiatric healthcare visits | 0.108949 | 6.9 |
|  | Days unemployed (year pre-treatment) | 0.045764 | 2.9 |
| SVM | Primary diagnosis: Panic disorder | 0.070988 | 22.9 |
|  | Income quintile | 0.050185 | 16.2 |
|  | Sex | 0.048993 | 15.8 |
|  | LSAS-SR at screening | 0.042389 | 13.7 |
|  | EQ-5D Pain/discomfort | 0.034056 | 11.0 |
|  | Pre-treatment symptom severity | 0.030879 | 10.0 |
|  | Prior dispensations of psychotropic medication | 0.016176 | 5.2 |
|  | Prior psychiatric healthcare visits | 0.008445 | 2.7 |
|  | Days unemployed (year pre-treatment) | 0.00741 | 2.4 |

*^a^Mean(|SHAP|) values are reported in the native output space used by each explainer (TreeExplainer for random forest and XGBoost, LinearExplainer for logistic regression, and KernelExplainer for SVM). Therefore, raw mean(|SHAP|) magnitudes are not directly comparable across model types.*

*^b^Normalized global SHAP importances for each final refit base learner, reported as mean absolute SHAP and as percentage of the total mean absolute SHAP across predictors included in the model. Logistic regression, XGB, and SVM include predictors retained by elastic net (n=9 each). Random forest was fit without elastic net and includes all predictors (n=56); the table displays only the top-10 predictors, hence SHAP% values do not sum to 100% for random forest.*
