# Supplementary material for: Prediction of Clinically Meaningful Improvement After Internet-Delivered Cognitive Behavioral Therapy for Depression and Anxiety Disorders: Machine Learning–Based Predictive Model Development and Temporal Validation Study
Source: J Med Internet Res. 2026 Aug 7;28:e100162. doi: 10.2196/100162 (PMC13451060; doi:10.2196/100162)
Supplement: Supplementary file 17 [file jmir-v28-e100162-s017.docx]

**PGS – clinically meaningful improvement associations significant at the unadjusted (nominal) level (*P*<0.05)**

| **Trait** | **UK Biobank field ID** | **OR [95% CI]** | ***p*-value** | ***p*-value adjusted^a^** |
| --- | --- | --- | --- | --- |
| Panic disorder | panic2019 | 1.25 [1.12, 1.38] | <0.001 | 0.014 |
| Age at first episode of depression | f.20433.0.0_res | 1.15 [1.04, 1.26] | 0.006 | 0.700 |
| Age at last episode of depression | f.20434.0.0_res | 1.13 [1.02, 1.24] | 0.015 | 0.789 |
| Family relationship satisfaction | f.4559.0.0_res | 1.12 [1.01, 1.23] | 0.026 | 0.864 |
| Thoughts of death during worst depression | f.20437.0.0_logistic | 1.11 [1.01, 1.22] | 0.039 | 0.952 |
| Happiness | f.4526.0.0_res | 1.10 [1.00, 1.21] | 0.047 | 0.952 |
| Obsessive compulsive disorder | ocd2018 | 0.90 [0.82, 1.00] | 0.041 | 0.952 |
| Number of full sisters | f.1883.0.0_res | 0.89 [0.81, 0.98] | 0.022 | 0.820 |
| Hearing difficulty/problems | f.2247.0.0_logistic | 0.89 [0.81, 0.98] | 0.018 | 0.789 |
| Frequency of tiredness / lethargy in last 2 weeks | f.2080.0.0_res | 0.89 [0.81, 0.98] | 0.018 | 0.789 |
| Ever had same-sex intercourse | f.2159.0.0_logistic | 0.89 [0.81, 0.98] | 0.016 | 0.789 |
| Family (siblings) history of Severe depression | 20111_12_logistic | 0.88 [0.80, 0.96] | 0.007 | 0.700 |

*PGS, polygenic score; OR, odds ratio*

*^a^p-value adjusted, p-value corrected at false discovery rate = 5% with the Benjamini-Hochberg procedure*
