# Supplementary material for: Prediction of Clinically Meaningful Improvement After Internet-Delivered Cognitive Behavioral Therapy for Depression and Anxiety Disorders: Machine Learning–Based Predictive Model Development and Temporal Validation Study
Source: J Med Internet Res. 2026 Aug 7;28:e100162. doi: 10.2196/100162 (PMC13451060; doi:10.2196/100162)
Supplement: Supplementary file 18 [file jmir-v28-e100162-s018.docx]

**Secondary analysis: Performance of phenotypic models predicting post-treatment score in ICBT (continuous outcome)^a^**

| **Model** | **RMSE** | **95% CI** | **MAE** | **95% CI** | **R^2^** | **95% CI** |
| --- | --- | --- | --- | --- | --- | --- |
| **Nested CV^b^** |  |  |  |  |  |  |
| Benchmark | 13.130 | [12.716, 13.543] | 10.354 | [10.055, 10.653] | 0.347 | [0.305, 0.388] |
| Logistic regression | 12.739 | [12.028, 13.450] | 10.014 | [9.479, 10.549] | 0.382 | [0.340, 0.424] |
| Random forest | 12.996 | [12.229, 13.762] | 10.318 | [9.766, 10.870] | 0.359 | [0.321, 0.396] |
| XGBoost | 12.956 | [12.241, 13.671] | 10.244 | [9.699, 10.790] | 0.361 | [0.320, 0.402] |
| SVM | 12.774 | [12.067, 13.482] | 10.050 | [9.257, 10.572] | 0.378 | [0.335, 0.422] |
| Soft voting ensemble | 12.743 | [12.016, 13.470] | 10.060 | [9.518, 10.602] | 0.382 | [0.343, 0.422] |
| Stacking ensemble | 12.740 | [12.025, 13.455] | 10.040 | [9.517, 10.564] | 0.383 | [0.342, 0.423] |
| **Holdout test set^c^** |  |  |  |  |  |  |
| Benchmark | 14.522 | [13.002, 16.053] | 11.198 | [10.251, 12.262] | 0.357 | [0.279, 0.437] |
| Logistic regression | 14.394 | [12.830, 15.927] | 10.995 | [9.993, 12.065] | 0.369 | [0.286, 0.442] |
| Random forest | 14.423 | [12.995, 15.957] | 11.007 | [10.047, 11.990] | 0.366 | [0.292, 0.439] |
| XGBoost | 14.380 | [12.914, 15.993] | 10.979 | [10.031, 11.991] | 0.370 | [0.292, 0.442] |
| SVM | 14.506 | [12.900, 16.045] | 11.059 | [10.032, 12.124] | 0.359 | [0.269, 0.434] |
| Soft voting ensemble | 14.270 | [12.789, 15.806] | 10.876 | [9.919, 11.908] | 0.380 | [0.301, 0.457] |
| Stacking ensemble | 14.287 | [12.774, 15.806] | 10.887 | [9.888, 11.922] | 0.378 | [0.298, 0.449] |

*ICBT, internet-delivered cognitive behavioral therapy; RMSE, root mean-squared error; MAE, mean absolute error; SVM, support vector machines*

*^a^On the 0–100 outcome scale*

*^b^Nested CV outer fold performance is reported with 95% CI to account for multiple‐imputation uncertainty (Rubin’s rules)*

*^c^Holdout test set performance is reported with 95% bootstrap CI to quantify sampling variability*
