# Supplementary material for: Prediction of Clinically Meaningful Improvement After Internet-Delivered Cognitive Behavioral Therapy for Depression and Anxiety Disorders: Machine Learning–Based Predictive Model Development and Temporal Validation Study
Source: J Med Internet Res. 2026 Aug 7;28:e100162. doi: 10.2196/100162 (PMC13451060; doi:10.2196/100162)
Supplement: Supplementary file 19 [file jmir-v28-e100162-s019.docx]

**Sensitivity analysis excluding patients with panic disorder**

|  | **LR** | **RF** | **XGBoost** | **SVM** | **Soft voting ensemble** | **Stacking ensemble** |
| --- | --- | --- | --- | --- | --- | --- |
| **Nested CV^a^** |  |  |  |  |  |  |
| AUC | 0.638 [0.606, 0.669] | 0.627 [0.613, 0.641] | 0.614 [0.588, 0.640] | 0.613 [0.582, 0.644] | 0.638 [0.612, 0.664] | 0.637 [0.612, 0.662] |
| BACC | 0.590 [0.562, 0.619] | 0.573 [0.559, 0.587] | 0.552 [0.539, 0.564] | 0.584 [0.554, 0.613] | 0.590 [0.564, 0.617] | 0.581 [0.558, 0.603] |
| F1 | 0.637 [0.608, 0.666] | 0.626 [0.612, 0.640] | 0.293 [0.238, 0.348] | 0.639 [0.602, 0.677] | 0.637 [0.606, 0.667] | 0.707 [0.688, 0.726] |
| Log loss | 0.667 [0.650, 0.684] | 0.664 [0.660, 0.668] | 0.704 [0.694, 0.713] | 0.659 [0.646, 0.672] | 0.663 [0.655, 0.672] | 0.654 [0.644, 0.665] |
| MCC | 0.181 [0.124, 0.239] | 0.147 [0.118, 0.175] | 0.132 [0.101, 0.163] | 0.169 [0.111, 0.228] | 0.181 [0.128, 0.234] | 0.185 [0.131, 0.240] |
| **Holdout test set^b^** | | | | | | |
| AUC | 0.693 [0.628, 0.755] | **Calibration curve of the recalibrated**  **LR model on the holdout test set** | | Total sample excluding patients with panic disorder:  n=1321 (training set: n=1058, holdout test set: n=263)  LR and soft voting performed equivalently on the primary metric (AUC) in nested CV. LR was taken forward to holdout evaluation based on parsimony, interpretability, and deployability.  Calibration was evaluated after retraining on the entire training set. Before single-pass evaluation on the holdout test set, the final LR model was recalibrated by 5-fold cross-validation on the training set, using Platt scaling (sigmoid). | | |
| BACC | 0.635 [0.575, 0.692] |  | |  |  |  |
| F1 | 0.622 [0.547, 0.688] |  |  |  |  |  |
| Log loss | 0.654 [0.623, 0.685] |  |  |  |  |  |
| MCC | 0.270 [0.151, 0.384] |  |  |  |  |  |
| Threshold***^c^*** | 0.584 |  |  |  |  |  |
| Sensitivity | 0.608 [0.521, 0.693] |  |  |  |  |  |
| Specificity | 0.662 [0.582, 0.742] |  |  |  |  |  |
| PPV | 0.637 [0.552, 0.721] |  |  |  |  |  |
| NPV | 0.633 [0.551, 0.713] |  |  |  |  |  |

*LR, logistic regression; RF, random forest; SVM, support vector machines; CV, cross-validation; AUC, area under the receiver operating characteristic curve; BACC, balanced accuracy; MCC, the Matthews correlation coefficient; PPV, positive predictive value; NPV, negative predictive value*

*^a^Nested CV outer fold performance is reported with 95% CI to account for multiple‐imputation uncertainty (Rubin’s rules)*

*^b^Holdout test set performance is reported with 95% bootstrap CI to quantify sampling variability*

*^c^Youden’s J threshold learned on the training set and applied unchanged to the holdout test set*
